# Here comes the sun: How optimization of photosynthetic light reactions can boost crop yields

**DOI:** 10.1111/jipb.13206

**Published:** 2022-02-26

**Authors:** Julia Walter, Johannes Kromdijk

**Affiliations:** ^1^ Department of Plant Sciences University of Cambridge Cambridge CB2 3EA UK; ^2^ Carl R Woese Institute for Genomic Biology University of Illinois Urbana‐Champaign Urbana Illinois 61801 USA

**Keywords:** bioengineering, crop improvement, electron transfer, light reactions, photosynthesis, photosystem, stress tolerance

## Abstract

Photosynthesis started to evolve some 3.5 billion years ago CO_2_ is the substrate for photosynthesis and in the past 200–250 years, atmospheric levels have approximately doubled due to human industrial activities. However, this time span is not sufficient for adaptation mechanisms of photosynthesis to be evolutionarily manifested. Steep increases in human population, shortage of arable land and food, and climate change call for actions, now. Thanks to substantial research efforts and advances in the last century, basic knowledge of photosynthetic and primary metabolic processes can now be translated into strategies to optimize photosynthesis to its full potential in order to improve crop yields and food supply for the future. Many different approaches have been proposed in recent years, some of which have already proven successful in different crop species. Here, we summarize recent advances on modifications of the complex network of photosynthetic light reactions. These are the starting point of all biomass production and supply the energy equivalents necessary for downstream processes as well as the oxygen we breathe.

## Graphical abstract

Improving photosynthesis is important for increasing future crop production. This review examines strategies to optimize protein complexes involved in photosynthetic light reactions in a range of plant/microalgal species; several of these strategies have been successfully transferred into crops, boosting yields and stress tolerance.

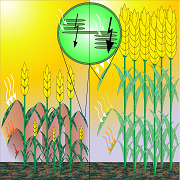

## INTRODUCTION—WHY DO WE NEED CROPS WITH INCREASED YIELDS?

**Figure 1:**
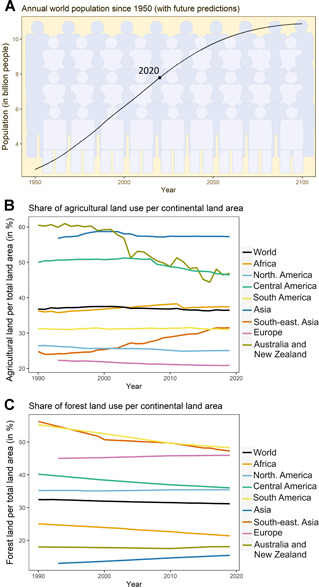
Global developments calling for solutions for improved crop yields (**A**) Annual world population (in billion people) recorded from 1950 to 2020 with future predictions from 2021 to 2100. (**B**) Agricultural land use development and (**C**) forest land development per continent over the past 30 years. The shares of annual agricultural land use and forested land (in %) per total land area was determined for each area. All data were obtained from the Food and Agriculture Organization (FAO) in October–December 2021. Agricultural land use includes both crop and pasture land. Timeseries data for Asia and Europe were started at 1993 to avoid the discontinuity in 1992 due to the end of the USSR. Groupings by continent or sub‐continent follow the FAO country groupings. The data for group “South America” also includes the Caribbean countries. The data for group “Asia” combines FAO country groups for central, eastern, southern, and western Asia.

Crops and farming have sustained human existence for more than 11,000 years (Murphy, 2007). The growing world population is currently projected to reach 10.87 billion people by the end of this century in 2100 (Figure 1A, data from the Food and Agriculture Organization [FAO, https://www.fao.org/faostat/]) and requires considerably increased food production. This is a major challenge as agricultural land becomes more and more limited. In many northern latitude countries, agricultural areas have not further expanded in the past 30 years or have even declined somewhat (Figure 1B, data from FAO, see also Ramankutty et al., 2018). However, in many tropical areas, agricultural land use has increased by up to 20% in the past 30 years, with concomitant decreases in forest area. Up to 40 hectares of forest are being cleared every minute to generate more arable land in order to produce more food and feed for animals. This has led to a more than 50% loss of rainforests to date (Figure 1C, data from FAO; FAO and UNEP, 2020).

**Figure 2:**
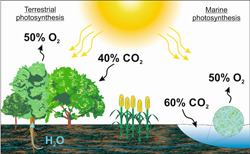
Schemes of terrestrial and marine photosynthesis Carbon dioxide (CO_2_) from the air and water (H_2_O) from the soil are taken up by land plants and converted into sugars and biomass using the light energy of the sun (according to the equation 6 CO_2 _+_ _6 H_2_O↔C_6_H_12_O_6 _+_ _6 O_2_). As a photosynthetic by‐product, molecular oxygen (O_2_) is released into the air. Percentages indicate the proportions of total sequestered CO_2_ and released O_2_ by terrestrial and marine photosynthesis (*source*: Food and Agriculture Organization https://www.fao.org/3/y0900e/y0900e06.htm, World Ocean Review).

Trees absorb and fix enormous amounts of solar radiation and carbon dioxide, making a major contribution to mitigate against global warming and climate change, and serve as reservoirs for the freshwater we drink. To tackle deforestation and preserve nature, alternative approaches need to be developed to improve productivity of the farmland that is currently available. One promising target is the chemical process that sustains all life on Earth, called oxygenic photosynthesis. Plants and photosynthetic micro‐organisms fix carbon dioxide from the atmosphere and convert it into sugars and organic biomass, using the absorbed light energy from the sun and water from the soil, and releasing molecular oxygen as a by‐product (Figure 2).

The predominant view up to two decades ago was that photosynthetic efficiency had reached its maximum capacity and could not be further improved in crops due to sink limitations, and the subject still sparks scientific debate (discussed in Long et al., 2006; Sinclair et al., 2019; Araus et al., 2021; Paul, 2021). However, studies simulating possible future increases in atmospheric carbon dioxide (CO_2_) concentrations have clearly indicated that photosynthesis can indeed be enhanced and result in improved crop yields (Ainsworth and Long, 2021), which has been further established over the years (Zhu et al., 2010; Parry et al., 2011; Gu et al., 2014; Long et al., 2015; Ort et al., 2015; Yin et al., 2015, 2021a, 2021b; Kromdijk and Long, 2016; Simkin et al., 2019; Wu et al., 2019).

In recent years, different strategies to improve crop yields have been proposed and reviewed, such as: (i) introducing photorespiratory bypasses (Betti et al., 2016; Hagemann and Bauwe, 2016; South et al., 2018; Eisenhut et al., 2019; López‐Calcagno et al., 2019; Maurino, 2019; Shen et al., 2019; Khurshid et al., 2020; Wang et al., 2020; Abbasi et al., 2021); (ii) introducing algal/cyanobacterial carbon concentrating mechanisms (McGrath and Long, 2014; Rae et al., 2017; Long et al., 2018; Atkinson et al., 2020; Hennacy and Jonikas, 2020; Chen et al., 2021; Rottet et al., 2021); (iii) introducing the C4 photosynthesis pathway into C3 plants (Ermakova et al., 2020, 2021a); (iv) improving mesophyll conductance (Hanba et al., 2004; Xu et al., 2019; Lundgren and Fleming, 2020; Ermakova et al., 2021c); (v) modifying metabolic processes (Rossi et al., 2015; South et al., 2019); and (vi) modifying circadian rhythms and introducing chronocultures (Steed et al., 2021). During the second half of the 20th century, the Green Revolution led to improved grain yields through conventional breeding techniques and improved pest/disease control. Nevertheless, photosynthesis still typically performs at a four‐ to five‐fold lower efficiency than its theoretical maximum (Long et al., 2015; Ort et al., 2015). Photosynthetic light use efficiency is a major determinant of the conversion efficiency of absorbed light energy into biomass. Only 50% of incident solar radiation (wavelengths between 400 and 740 nm) can be actively used to drive photosynthesis. Further energy losses occur due to light reflectance from the leaf, light absorption by nonphotosynthetic pigments, dissipation of excess light energy as heat, thermodynamic limits, carbohydrate biosynthesis, photorespiration, and respiration. This leaves a theoretical maximum of about 5% of total irradiance that is converted into biomass. However, in the field, photosynthetic efficiencies normally only reach 1%–2% on the individual plant level because of light saturation of the photosynthetic machinery at about 25%–50% of full sunlight and activity of energy‐dissipating photoprotective mechanisms at higher light intensities (Long et al., 2006; Zhu et al., 2008, 2010). At the canopy level this results in photosynthetic efficiencies of about 2.2% under well‐managed conditions (Yin and Struik, 2015). Calculating these theoretical efficiencies highlights the importance of crop modeling to consider further routes for crop improvement (Wu et al., 2019; Yin et al., 2021a, 2021b). Strategies to overcome these limitations include optimization of the canopy and leaf architecture (Tholen et al., 2012; Drewry et al., 2014; Mathan et al., 2016; Song et al., 2013, 2016; Xiao et al., 2016; Slattery and Ort, 2021) as well as the photosynthetic light‐dependent and light‐independent reactions. While the photochemical light‐dependent reactions involve harvesting of excitation energy from sunlight to produce the energy carriers nicotinamide adenine dinucleotide phosphate (reduced form) (NADPH) and adenosine triphosphate (ATP), in the light‐independent reactions these energy carriers are then used to fix carbon dioxide via the enzyme ribulose‐1,5‐bisphosphate carboxylase/oxygenase (Rubisco) into C3 sugars and regenerate the Rubisco substrate ribulose‐1,5‐bisphosphate (RuBP; Calvin–Benson–Bassham cycle). Both Rubisco activity and the rates of regeneration reactions have been targeted for improvement, which have proven very successful in recent years (Lefebvre et al., 2005; Kurek et al., 2007; Kumar et al., 2009; Rosenthal et al., 2011; Parry et al., 2013; Whitney et al., 2015; Driever et al., 2017; Salesse‐Smith et al., 2018; Simkin et al., 2015, 2017a, 2019; Scafaro et al., 2019a; López‐Calcagno et al., 2020; Iñiguez et al., 2021), with more promising research under way (Scafaro et al., 2019b; Degen et al., 2020).

This review focuses on the photosynthetic light‐dependent reactions, the optimization of which is relevant to improving both C3 and C4 photosynthesis in plants and photosynthetic micro‐organisms (Leister, 2012; Ruban, 2015; von Caemmerer and Furbank, 2016; Cardona et al., 2018; Simkin et al., 2019; Batista‐Silva et al., 2020; Ermakova et al., 2021b; Sales et al., 2021; Santos‐Merino et al., 2021). Modeling studies have suggested that improving the quantum yield and electron transport capacity have a greater potential for increasing the productivity of crops than other photosynthetic mechanisms, such as improving Rubisco activity (Gu et al., 2014; Yin and Struik, 2015, 2021a; Wu et al., 2019). In the past couple of years, numerous novel approaches to improve photosynthetic light reactions have been reported. This review presents a synthesis and highlights the potential of these approaches to boost crop yields.

## THE “SOLAR PANELS” OF THE PLANT CELL—THE LIGHT REACTIONS OF PHOTOSYNTHESIS

Solar panels are an increasingly popular choice for generating “home‐made” sustainable energy and circumvent the use of fossil fuels (International Energy Agency, 2021). The process of light energy conversion into electricity, called photovoltaic effect, has been translated into solar cells with light conversion efficiencies of around 20%–50% (Geisz et al., 2020). A similar effect can be observed in nature in photosynthesizing organisms. Here, the light‐harvesting complexes together with the photosystems act in series to absorb energy from sunlight and fuel electron transfer and subsequent redox reactions in the thylakoid membranes, resulting in the generation of chemical energy which fuels the production of biomass. However, the photosynthetic light‐to‐biomass conversion efficiency is more difficult to estimate than the light‐to‐electricity conversion efficiency in solar cells. Nevertheless, efforts have been made to determine the theoretical photosynthetic efficiency in plants on the individual plant level as 4.6% and 6.0% (Zhu et al., 2010) for C3 and C4 plants, respectively. However, actual photosynthetic efficiencies can be as little as 1%–3.5%/4.3% in C3/C4 plants (Zhu et al., 2010; Blankenship et al., 2011). But how exactly does the light‐harvesting “solar panel” of the plant cell operate when a photon hits the leaf and initiates photosynthesis? And how has it developed during the course of evolution?

**Figure 3:**
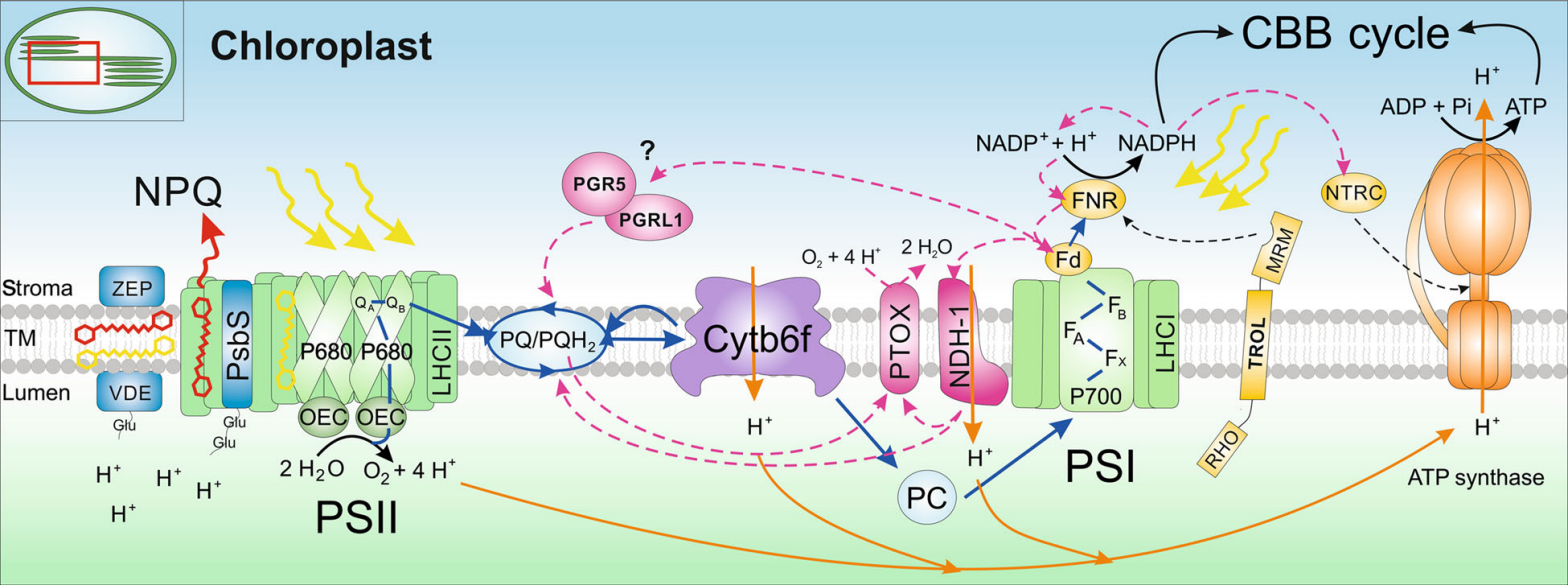
Overview of light reactions in the thylakoid membrane In the thylakoid membrane system inside the chloroplast, two pigment–protein photosystems (PSII and PSI) operate in series in order to generate the energy equivalents nicotinamide adenine dinucleotide phosphate (reduced form) (NADPH) and adenosine triphosphate (ATP). Absorbed excitation energy is channeled by light‐harvesting complexes LHCII and LHCI toward the reaction centers of both photosystems (P680 in PSII and P700 in PSI), where an electron is liberated and passed along several electron acceptors down the linear electron transfer (LET) chain (blue arrows). Downstream of PSII, the plastoquinone/plastoquinol (PQ) pool transfers electrons to the cytochrome b6f complex (cyt b6f) and further to PSI via plastocyanin (PC). In PSI, several iron‐sulfur clusters (F_x_, F_A_, F_B_) are the primary electron acceptors which then reduce ferredoxin (Fd). Lastly in LET, the Fd‐NADP^+^ reductase (FNR) is released from its possible anchor thylakoid rhodanase‐like (TROL) protein, which contains a rhodanese‐like motif (RHO) in the lumen and a membrane recruiting motif (MRM) in the stroma, and regenerates NADPH by oxidation of Fd. Simultaneously in PSII, oxidized P680^+^ is reduced by an electron deriving from the splitting of water (H_2_O) at the oxygen‐evolving complex, which also releases molecular oxygen (O_2_) and protons (H^+^). Protons are also transported across the thylakoid membrane by cyt b6f and the NADH dehydrogenase‐like 1 (NDH‐1) complex (orange arrows) in order to fuel ATP production at the ATP synthase complex. Both NADPH and ATP are then metabolized in the Calvin–Benson–Bassham (CBB) cycle for carbon fixation. In case of overexcitation of the LET chain, AET routes are activated downstream of PSI (magenta dashed arrows), including cyclic electron transfer via the Proton Gradient Regulation 5 (PGR5)/PGR5‐like photosynthetic phenotype 1 (PGRL1) or the NDH‐1 complexes. NDH‐1 also diverts excess electrons to the Plastid Terminal Oxidase PTOX, which reduces O_2_ to H_2_O. Photoprotection via qE‐type nonphotochemical quenching (NPQ) involves the PSII subunit S protein PsbS, which senses the acidification of the lumen upon high light exposure via protonatable residues and initiates the rearrangement of the LHCII complexes, thus inducing the dissipation of excess excitation energy or NPQ. In parallel, the xanthophyll cycle is also activated by the lumen pH, inducing the reversible conversion of violaxanthin (yellow pigment) into antheraxanthin and zeaxanthin (red pigment) in light via the violaxanthin de‐epoxidase (VDE). In dark, the xanthophyll cycle is then reversed by zeaxanthin epoxidase (ZEP).

As the name suggests, light‐harvesting complex (LHC) proteins absorb light energy from the sun. The LHC proteins are located in the thylakoid membranes in close proximity to the photosystems PSII and PSI and act as a funnel, channeling the absorbed light energy, also called excitation energy, toward the photosystems' reaction center chlorophylls P680 (PSII) and P700 (PSI). There, electrons become excited by reaching a higher energy level (termed “charge separation”) and move toward electron acceptors, thus initiating a series of electron transfers in the thylakoid membrane between the two photosystems. Charge separation in PSII leads to oxidized P680^+^ and reduction of the stable electron acceptors plastoquinone (PQ) A and B (Q_A_ and Q_B_, PQ pool) via the unstable intermediate pheophytin. P680^+^ is a very strong oxidant and extracts an electron from water, which is split into protons (H^+^) and molecular oxygen (O_2_) at the Oxygen‐Evolving Complex in PSII. Further down the line, doubly reduced Q_B_ accepts two H^+^ from the stroma, forming the mobile electron carrier plastoquinol (PQH_2_), which passes on the electron to the membrane‐embedded cytochrome b6f complex. From there, the electron either travels back to the PQ pool via the Q‐cycle or further toward the electron gap in PSI (P700^+^) via a mobile carrier in the thylakoid lumen, which is called plastocyanin. Within PSI then, excited electrons released from P700 using harvested light energy, are accepted by phylloquinone followed by electron transfer via three iron‐sulfur (Fe‐S) clusters to ferredoxin (Fd) and the protein Fd‐NADP^+^‐reductase (FNR) which regenerates NADP^+^ to the reducing agent NADPH. At the same time, the ultimate energy carrier ATP is produced upon acidification of the thylakoid lumen. During linear electron transfer (LET), a H^+^ gradient across the thylakoid membrane is established. This electrochemical force (proton motive force—pmf) drives ATP synthesis via the membrane‐spanning ATP synthase complex, which generates ATP from adenosine diphosphate (ADP) and inorganic phosphate (P_i_) on the stromal side. The NADPH and the ATP produced by photosynthetic electron transport are essential to drive the Calvin–Benson–Bassham cycle for atmospheric CO_2_ fixation and the production of sugars used for biomass biosynthesis, and for other metabolic processes in the chloroplast (for a review see Stirbet et al., 2019; Figure 3).

### The more sunlight, the better? How photoprotective mechanisms safeguard the light reactions from excessive sunlight

In nature, sunlight is a very variable resource. On a sunny day, plants can experience fluctuations in light exposure and spectral features when clouds cover the sun, for instance, or other plants/leaves move in the wind and shade the canopy below. Thereby, blue and red wavelengths are absorbed by the upper canopy, depleting the light of wavelengths that can be captured by the LHC proteins and drive photosynthesis in the lower canopy. Dynamic light conditions occur on a seasonal level, a daily level, and as cloud‐ and sunflecks, which can fluctuate rapidly and last seconds to minutes (Morales and Kaiser, 2020).

Harvested light energy does not always lead to electron transfer but can also be dissipated via other routes. If the sunlight is too strong (termed “high light”), it can be harmful to the plant. Photosynthesis has several rate‐limiting steps, such as the regeneration of the Rubisco substrate RuBP as electron sink in the Calvin–Benson–Bassham cycle as well as the activity of the cytochrome b6f complex and the ATP synthase in the light reactions (Farquhar et al., 1980; Price et al., 1998; Hajirezaei et al., 2002; Sage et al., 2008; Rott et al., 2011; Yamori et al., 2011; Hasan and Cramer, 2012; Magyar et al., 2018). These bottlenecks can result in a jam of electrons in the thylakoid membrane when the capacity of light‐harvesting exceeds the capacity of CO_2_ fixation. Under these conditions, the formation of chlorophyll triplet states from singlet excited chlorophylls increases. Triplet chlorophyll can readily react with molecular oxygen and form the harmful reactive oxygen species (ROS) singlet oxygen. This highly reactive state of oxygen can specifically damage the photosynthetic protein complexes in the thylakoid membrane, oxidize plasma membrane lipids and react with nucleic acids (Krieger‐Liszkay, 2005; Di Mascio et al., 2019; Khorobrykh et al., 2020). Particularly, the PSII core protein D1 is prone to oxidative photodamage upon long‐term high light exposure, causing a transient downregulation of PSII efficiency, termed photoinhibition of PSII (Aro et al., 1993; Long et al., 1994; Murata et al., 2007; Guidi et al., 2019). Since plants are sessile organisms, they are not able to change location to avoid unfavorable conditions and have developed numerous photoprotective mechanisms during the course of evolution. One such mechanism is via nonphotochemical dissipation of excess absorbed energy as heat (nonphotochemical quenching [NPQ]).

**Figure 4:**
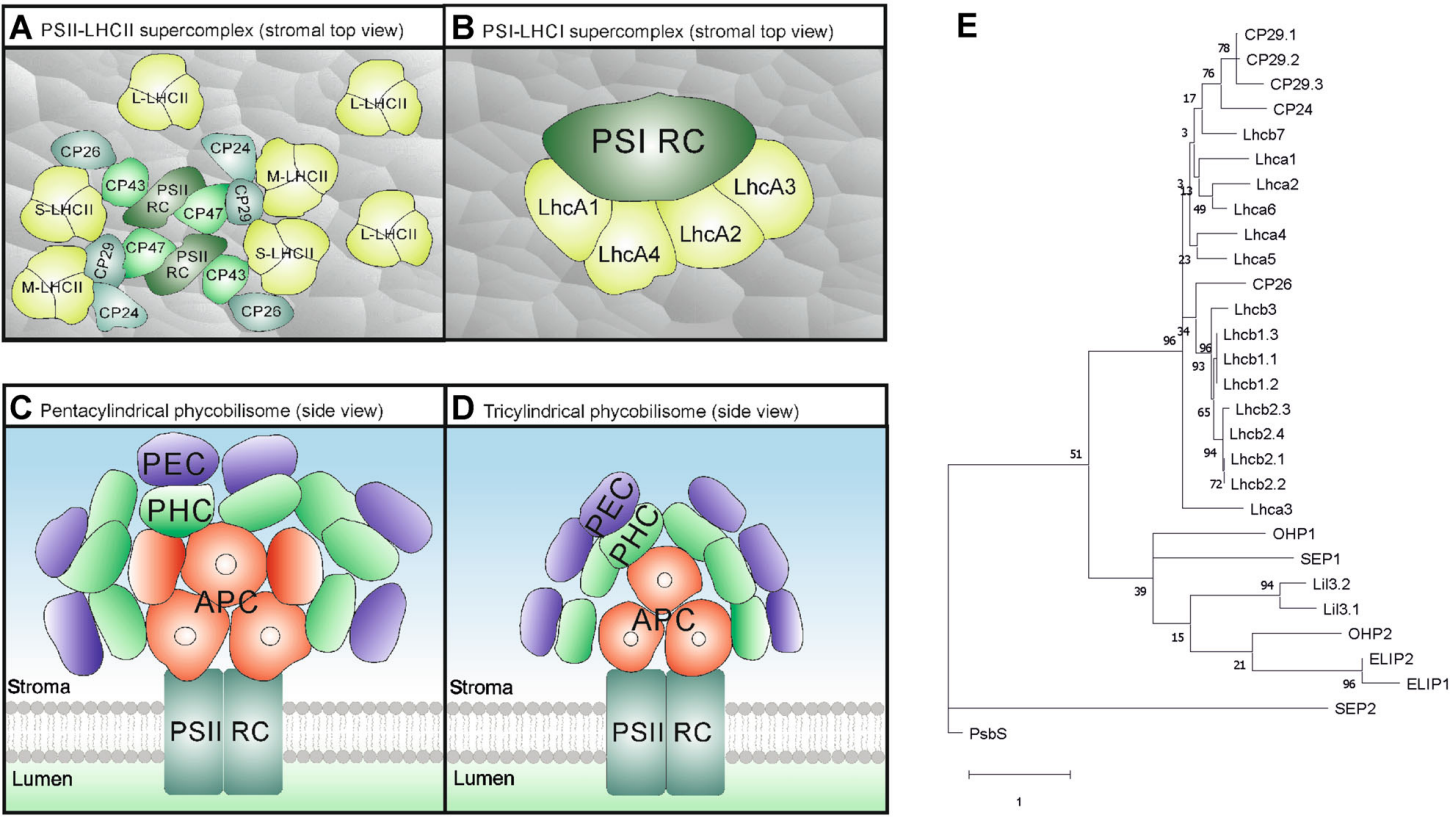
Arrangements of photosystems with light‐harvesting complexes (LHC) in higher plants and cyanobacteria in the thylakoid membranes Top views of the pigment–protein photosystem II (PSII)‐LHCII (**A**) and PSI‐LHCI (**B**) supercomplex core components in higher plants (recreated from Protein Data Bank (PDB) entries 5MDX and 2WSC). The dimeric PSII core consists of the reaction center (RC) proteins D1 and D2 and the core proteins CP43 and CP47. The minor antennae CP29/CP24 (heterodimer) and CP26 connect moderately and strongly bound M‐ and S‐LHCII trimers to the PSII RC via CP47 and CP43, respectively. Loosely bound L‐LHCII trimers are often detached from the supercomplex. In monomeric PSI, the RC is surrounded by LhcA1‐4 in a fan‐like fashion, with LhcA1 and A4 and LhcA2 and A3 forming heterodimers. In contrast, cyanobacterial light‐harvesting antennae are not embedded within the thylakoid membrane but are attached to the core proteins on the stromal surface. These large pigment‐protein complexes are called phycobilisomes (PBS) and come in different shapes. Pentacylindrical PBS are predominantly present in filamentous cyanobacteria and consist of five allophycocyanin (APC) core cylinders (red) from which eight phycoerythrocyanin (PEC; blue)/phycocyanin (PHC; green) rods radiate (**C**). The colors represent the wavelengths the different PBS discs absorb upon binding phycocyanobilin pigments, inducing energy transfer from PEC→PHC→APC→PSII RC. Unicellular cyanobacteria mostly contain tricylindrical PBS with three APC core cylinders and up to six rods (**D**). The structures were generated from PDB entries 7EYD (*Anabaena* sp. PCC 7120) and 7EXT (*Synechococcus* sp. PCC 7002), respectively. (**E**) Phylogenetic tree of *Arabidopsis thaliana* LHC family proteins generated in MEGAX64. Protein sequences were aligned by MUSCLE with the UPGMA cluster method. The tree was built using Maximum Likelihood as statistical method, in conjunction with the bootstrap method (500 replications), the rtREV with Freqs. (+F) model, Gamma Distributed rates with Invariant Sites (G + I; five discrete gamma categories), partial deletion of gaps (95% cutoff) and the Nearest‐Neighbor‐Interchange heuristic model.

In addition to photochemistry and NPQ, excited chlorophylls can also return to the ground state via fluorescence, that is, emission of a red‐shifted photon. While fluorescence emission is not an appreciable energy flux, analysis of fluorescence quenching allows estimation of energy dissipation via photochemistry and NPQ. NPQ measured by fluorescence quenching analysis is a collective term that includes several different components for the avoidance responses of photodamage. These actually do not dissipate excess energy as heat but aim at decreasing light absorption and optimizing electron distribution, such as chloroplast photorelocation, redistribution of LHCII between the photosystems (state transitions) and photoinhibitory break‐down of D1 in PSII (for a comprehensive overview and depiction see Malnoë, 2018; Messant et al., 2021). Energy dissipation in the LHCs via NPQ consists of different components with contrasting response times. The fastest component of actual heat dissipation mechanisms is called energy‐dependent quenching (qE) (Wraight and Crofts, 1970, reviewed in Ruban, 2016, depicted on the left‐hand side in Figure 3). qE is triggered by lumen acidification, with the sensitivity of the response highly dependent on the PSII subunit S protein (PsbS) in higher plants. This protein is considered the main player in the induction of photoprotective mechanisms and acts as a pH sensor in the thylakoid membrane. Upon illumination and thus acidification of the thylakoid lumen, PsbS undergoes a conformational change and activates quenching of excess absorbed light energy. A very likely location of the active quenching site in land plants are the LHC antenna proteins, which are involved in light‐harvesting as well as photoprotection of the photosystems' reaction centers. LhcA1‐4 proteins form heterodimeric antennae around PSI, whereas LhcB1‐3 assemble either into strongly PSII‐bound S‐homotrimers of LhcB1 or moderately and loosely PSII‐bound M‐ and L‐heterotrimers of LhcB1‐3. These major antennae of LHCII trimers are connected to the PSII core proteins via the monomeric minor antennae LhcB4‐6. While LhcB4 (CP29) and LhcB6 (CP24) form heterodimers, connecting the M‐LHCII trimers to the PSII core via the CP47 protein, LhcB5 (CP26) connects the S‐LHCII trimers to the PSII core via the CP43 protein, thus channeling the absorbed sunlight energy from the LHCII trimers to the PSII reaction center via the monomeric antenna proteins (Figure 4A,B). Although the exact NPQ mechanisms are not known yet, it seems clear that PsbS dimers sense the change in lumenal pH upon light exposure with two protonatable lumen‐exposed glutamate residues per monomer (Li et al., 2004). Subsequently, the rearrangement of the LHCII antennae is initiated, potentially via PsbS monomerization and interactions with the CP29 and LhcB1 proteins. In a putative model for qE, conformational changes are also induced at the supercomplex level, where M‐LHCII trimers are released from the PSII‐LHCII supercomplexes for the formation of LHCII aggregates in the thylakoid membrane, forming the putative quenching site Q1 (Bergantino et al., 2003; Teardo et al., 2007; Betterle et al., 2009; Miloslavina et al., 2011; Dall'Osto et al., 2017; Kress and Jahns, 2017). Besides the acidification of the lumen pH, another prerequisite of qE is the activation of the reversible xanthophyll cycle and binding of the xanthophyll zeaxanthin to major and minor LHCII proteins (Niyogi et al., 1997; Bassi and Caffarri, 2000; Ballottari et al., 2012). Under dark and low light conditions, LHCII proteins predominantly bind violaxanthin, whereas upon exposure to high light, violaxanthin is released into the thylakoid membrane, converted into zeaxanthin via the intermediate xanthophyll antheraxanthin by the enzyme violaxanthin de‐epoxidase (VDE), and reinserted to induce NPQ and protect the cell components from oxidative stress (Havaux et al., 2007; Johnson et al., 2007; Dall'Osto et al., 2010). Zeaxanthin is reconverted into violaxanthin by the stromal enzyme zeaxanthin epoxidase (ZEP) upon light‐to‐dark transitions. Zeaxanthin is also involved in a PsbS‐independent NPQ mechanism, called qZ (zeaxanthin‐dependent quenching) for which induction and relaxation are correlated with zeaxanthin formation and depletion on a time scale of several minutes (Dall'Osto et al., 2005; Nilkens et al., 2010). Whereas further long‐term quenching forms were previously collectively termed qI (inhibitory quenching), this parameter is currently subject to further molecular dissection based on the factors involved. For example, a long‐term form of NPQ (in the range of hours), called qH, has recently been discovered to occur in the LHCII trimers, involving the plastid lipocalin LCNP and its regulators SOQ1 and ROQH1 (Malnoë et al., 2018; Amstutz et al., 2020; Bru et al., 2020, 2021; Yu et al., 2021).

In addition to NPQ, several alternative electron transfer (AET) routes become active in response to stress in order to prevent the LET chain from overreduction. To maintain an optimal ATP/NADPH ratio for metabolic processes under such conditions, electrons are rerouted from Fd back to the PQ pool potentially via the Proton Gradient Regulation 5 (PGR5)/PGR5‐like photosynthetic phenotype 1 (PGRL1) complex or the NADH dehydrogenase‐like (NDH) complex, thus driving cyclic electron transfer (CET) around PSI and sustaining ATP synthesis (Ma et al., 2021). The membrane‐embedded NDH‐1 complex not only reduces the PQ pool for CET with electrons deriving from NADPH via FNR and Fd (Mulo and Medina, 2017) but also transfers protons into the thylakoid lumen for activation of the ATP synthase (Peltier et al., 2016). NDH‐1 is also proposed to be involved in a respiratory pathway inside the chloroplasts, called chlororespiration, which includes reoxidation of the PQ pool and reduction of oxygen to water by the membrane protein Plastid Terminal Oxidase (PTOX) as an alternative electron sink. PTOX was hypothesized to have a dual function, depending on the prevalent light intensity and thus the redox state of the PQ pool/lumen pH (Yu et al., 2014; Feilke et al., 2016; Krieger‐Liszkay and Feilke, 2016). Current models suggest that under high light conditions, PTOX associates with the thylakoid membrane and is accessible for its substrate plastoquinol (PQH_2_), hence oxidizing the over‐reduced PQ pool, using oxygen as an electron acceptor. However, in a side reaction, superoxide and hydroxyl radicals (ROS) are produced which could lead to photoinhibition of the photosystems if not scavenged properly by other antioxidant systems (Sun et al., 2017). Under nonsaturating light, on the other hand, it was demonstrated that PTOX can theoretically act as an extra electron sink and keep the PQ pool more oxidized and protected from photoinhibition, although its expression levels are extremely low under this condition, avoiding competition with LET (Feilke et al., 2016). However, this “antioxidant” feature is important under dynamic light conditions (Nawrocki et al., 2019b). In case superoxide is still produced at PSI, it is rapidly reduced to water by the enzymes superoxide dismutase and ascorbate peroxidase (Mehler reaction; Mehler, 1951). This reaction is part of the water–water cycle, referring to electron flow from the splitting of water at PSII to the generation of water at PSI, and acts as an alternative electron sink to increase the ATP:NADPH ratio under stress conditions when more ATP is required (Asada, 2000). Instead of stimulating ATP biosynthesis, the ATP:NADPH ratio can also be adjusted by removing NADPH either through enhanced uptake by metabolic processes, such as fatty acid production, or through transport into mitochondria via the malate shuttle (for a recent review see Dao et al., 2021).

### Evolution and structure of the LHC protein family

Throughout evolution, the main components of the photosynthetic machinery have remained well‐conserved in the plant tree of life, ranging from single cell blue–green algae (called cyanobacteria) to higher plants (Nelson and Yocum, 2006). In Archean times probably more than 3.5 billion years ago, a homodimeric photosystem reaction center diverged into Type II and Type I reaction centers. Type II reaction centers then further diversified into ancestral heterodimeric water‐splitting and nonwater‐splitting systems, giving rise to oxygenic and anoxygenic photosynthesis, respectively. Cyanobacteria are the first known organisms to employ oxygenic photosynthesis, having emerged about three billion years ago, releasing molecular oxygen into the atmosphere as a by‐product of splitting water molecules at PSII. Consequently, during the “Great Oxidation Event” 2.4 billion years ago, the reduced, high CO_2_ atmosphere was converted into an oxidized, low CO_2_ atmosphere—as we know it today—allowing the development of all oxygen‐breathing organisms (Sánchez‐Baracaldo and Cardona, 2020). Cyanobacteria are deemed the evolutionary predecessors of the photosynthetic chloroplast organelles in eukaryotes. Through an endosymbiotic event in which a heterotrophic eukaryotic α‐proteobacterium engulfed an autotrophic cyanobacterium hundreds of million years ago, the cyanobacterium integrated into the host cell and slowly developed into the chloroplast over time, leading to the generation of green algae and plant cells (Gould et al., 2008; McFadden, 2014).

However, one major difference in the physiology of the photosynthetic machinery between cyanobacteria and plants is the evolution of the light‐harvesting antenna complexes. While in green algae and plants, the pigment–protein antennae are embedded in the thylakoid membrane adjacent to the photosystems, in cyanobacteria, red algae and glaucophytes, they sit in a fan‐like fashion on top of the photosystems on the stromal side of the thylakoid membrane (Figure 4C,D). These phycobilisomes (PBS) are large complexes and comprise phycobiliproteins that can bind several types of linear phycobilin pigments. The standard cyanobacterial PBS structure consists of allophycocyanin core cylinders, from which rods composed of phycocyanin and phycoerythrocyanin/phycoerythrin radiate. Depending on which phycobiliprotein the pigments are bound to, they can absorb light of different wavelengths. The distal part of the rod absorbs short wavelengths in the blue–green range with a maximum at 570 nm. The captured energy is then transferred through the rods toward the PBS core, which absorbs longer wavelengths of red light (maximum at 650 nm) and passes on all energy to the PSII reaction center. Composition and length of the rods can be adjusted according to the prevalent light conditions (Bryant, 1982; Chang et al., 2015; Stadnichuk et al., 2015; Tang et al., 2015; Green, 2019; Bag, 2021). But “what happened to the phycobilisome?” (Green, 2019) and how did the LHC protein family eventually evolve?

With the evolution of the chloroplast, many photosynthetic genes from the ancestral cyanobacterium were transferred and incorporated into the nucleus of the eukaryotic host cell. The genes for the assembly of the PBS and the associated orange carotenoid protein (OCP) for photoprotection (Muzzopappa and Kirilovsky, 2020) were likely lost, possibly due to their large size and high nitrogen requirement for protein assembly. Cyanobacteria also contain high light‐inducible proteins (HLIPs), which are considered the evolutionary progenitors of the LHC protein family. These HLIPs are small single‐helix transmembrane proteins that bind chlorophyll a and the carotenoid β‐carotene and function in assembly and photoprotection of PSII and chlorophyll metabolism in cyanobacteria (Komenda and Sobotka, 2016; Tibiletti et al., 2018). Upon endosymbiosis of cyanobacterium and eukaryote, HLIP genes were transferred into the nucleus of the eukaryote and are still present in the plant genome as one‐helix proteins (OHPs; Figure 4E). Through acquisition of additional transmembrane domains, internal gene duplications and loss of helices, two‐helix stress‐enhanced proteins (SEPs) and light‐harvesting‐like (Lil) proteins, the nonpigmented four‐helix protein PsbS, as well as the big group of three‐helix LHC proteins (including early light‐inducible proteins [ELIPs]), respectively, developed especially in the green lineage of chlorophyta, ranging from green algae to higher plants (Green, 2019; Bag, 2021). A special case is the evolution of the presumably first LHC‐like protein group of LHCSR proteins and the “younger” PsbS protein. In the case of PsbS, pH sensing and the active quenching site are located on different proteins, whereas in LHCSR both mechanisms are combined in one protein. While photoprotection in green algae mainly relies on LHCSR proteins, in mosses, both LHCSR and PsbS proteins are active. PsbS development and relocation of the active quenching site to other protein complexes was likely an adaptation process to more challenging light conditions when plants started to conquer terrestrial land. This would also explain why LHCSR proteins are completely absent in land plants (Pinnola, 2019).

Furthermore, it is believed that CP29 was the first LHCII protein to evolve due to its presence in taxonomically diverse classes of green algae. This points to the existence of an ancestral CP29 gene before the diversification of the green lineage, followed by the rise of CP26 and ancestral major antenna proteins LhcBM in green algae through gene duplications and DNA crossovers of SEP genes. In contrast, CP24 seems to be the latest addition to the LHC protein family, being present in land plants only (Koziol et al., 2007). Even though the protein sequence similarities are only 20%–40%, the overall protein structures with three membrane‐spanning domains and conserved chlorophyll a/b‐binding sites are mostly uniform across the major and minor LHCII proteins (Jansson, 1999; Ballottari et al., 2012). In addition to chlorophylls, they also bind three to four carotenoids (xanthophyll pigments: lutein, violaxanthin, zeaxanthin, neoxanthin; and β‐carotene) to designated binding sites (Jahns and Holzwarth, 2012).

## IMPROVING CROP YIELDS THROUGH OPTIMIZATION OF PHOTOSYNTHETIC LIGHT REACTIONS

### Decreasing antenna size to increase biomass yield

**Figure 5:**
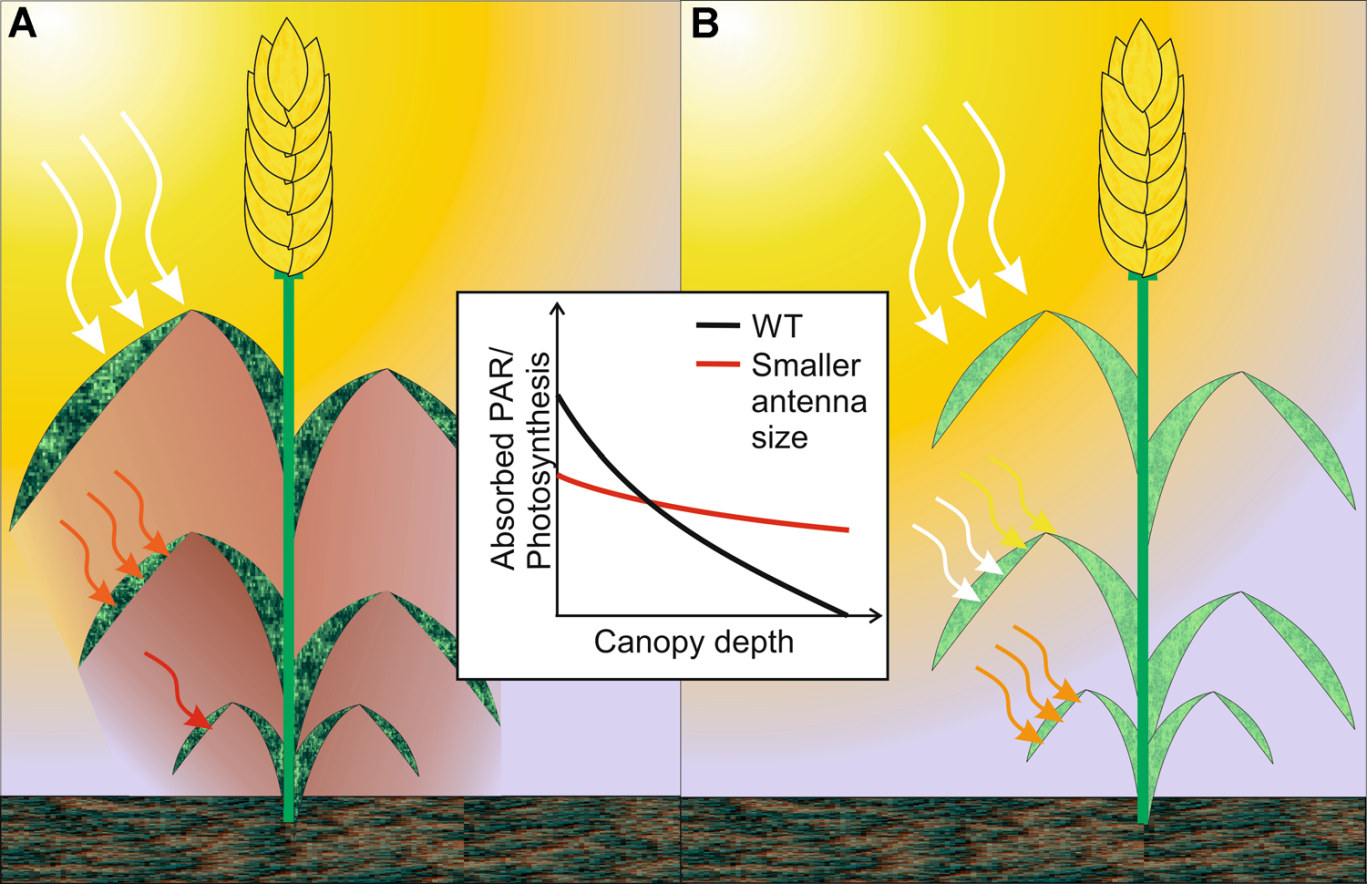
Light distribution across the plant canopy between wild type (A) and mutants with decreased light‐harvesting complex (LHC) antenna sizes (B) (**A**) In a dense crop canopy, light distribution across the leaves of the plant is very uneven because of shading effects from other leaves. Leaves of the upper canopy closest to the light source absorb most of the photosynthetic active radiation (PAR) and deplete the light in the lower canopy of its PAR, resulting in a strong decrease of photosynthesis with increasing canopy depth (see insert). (**B**) Introduction of mutants with a decreased cross‐section of the LHC antennae allows PAR to travel deeper into the crop canopy, as the fractional PAR absorption of leaves closest to the light source has decreased. This approach may marginally decrease photosynthesis of the light‐exposed layers but improves light distribution across the entire canopy, thus improving overall photosynthesis in the otherwise shaded layers and the entire plant (see insert).

In the previous sections, we described the functions of the light‐harvesting antenna complexes in the photosynthetic thylakoid membrane on the level of an individual leaf. However, what might be beneficial for an individual plant/leaf might not necessarily be in favor for the entire population/organism, respectively. As mentioned above, photosynthesis has several rate‐limiting steps, which inhibit electron transfer even when the energy input is maximized, causing photodamage of the photosynthetic machinery. In a typical crop canopy, leaves at the top would catch most of the actinic sunlight for the activation of photosynthesis, while light incident on the leaves below will be largely composed of wavelengths that are poorly absorbed by the LHC antennae and will be too low in intensity to drive high rates of photosynthesis (Figure 5). Thus, in an optimal case, leaves in the upper canopy would sacrifice maximum light absorption to increase light penetration to leaves in the lower canopy to sustain efficient photosynthesis throughout the plant. However, this is a difficult undertaking, both in traditional plant breeding as well as with genetic engineering. Plant breeders would normally select for traits that are beneficial for the individual plant, rather than for the entire population. For that reason, plants with a light green color or upright leaves were selected against in the past, signifying lower photosynthesis rates and light absorption, respectively, on an individual plant level, even though these traits could be favorable in a plant community to allow better light distribution across the entire canopy. On the other hand, plant populations with traits beneficial for the community are often invaded by individual plants with more competitive features. In terms of light interception, examples could be taller plant height, more horizontal leaf angles or bigger leaves (Anten and Vermeulen, 2016). These invaders then have an advantage in obtaining resources over the plant community, resulting in poorer crop yields at the stand level. With regard to genetic engineering, it is possible to independently modify traits in different plant tissues, but it is more challenging to independently optimize traits in individual leaves, for instance. Nevertheless, scientists have developed a range of novel approaches to tackle this problem and reduce the LHC antenna systems for better transmittance of sunlight through plant canopies (Ort et al., 2011). Similar approaches have also been generated for microalgal/cyanobacterial liquid mass cultures in photobioreactors, which are easier and faster to manipulate. Some successful strategies could have the potential of being translated from photosynthetic micro‐organisms into crop species in the future, and are therefore briefly described hereafter.

Microalgae and cyanobacteria have gained more and more attention as potential production platforms for biotechnological, pharmaceutical, and nutraceutical compounds over the past decades due to their photosynthetic ability and have many advantages over plant‐based bioengineering and production (reviewed in the past 3 years by Benedetti et al., 2018; Khan et al., 2018; Rizwan et al., 2018; Metsoviti et al., 2019; Naruka et al., 2019; Mutanda et al., 2020; Zahra et al., 2020; Dhandayuthapani et al., 2021; Khalifa et al., 2021). High production costs are currently the major drawback owing to a low light‐to‐biomass conversion rate inside the water column. Hence, several attempts have been made in different algal and cyanobacterial species to reduce the optical cross‐section of LHCII antennae to allow better light penetration into the water column of mass production bioreactors (Melis et al., 1999; Mussgnug et al., 2007; Kosourov et al., 2011; Ort et al., 2011; Cazzaniga et al., 2014; Kirst and Melis, 2014; De Mooij et al., 2015, 2016; Shin et al., 2016; Dall'Osto et al., 2019; Hu et al., 2020; Vecchi et al., 2020). In 2002, Polle and coworkers targeted the unicellular green alga species *Chlamydomonas reinhardtii* and *Dunaliella salina* for proof of this concept through DNA insertional and chemical‐induced mutations (Polle et al., 2002). Screening for mutants with reduced antenna size but increased photosynthetic performance revealed several genes that could be of interest for manipulation in order to maximize algal biomass or hydrogen production. This list of candidate genes predominantly included genes involved in the biosynthesis of chlorophylls and carotenoids and mutants had about 20%–50% smaller light‐harvesting antennae, mainly affecting PSII‐associated complexes. In a chl b‐less mutant, photosynthetic productivity could be increased two‐fold, although lower maximum PSII quantum efficiencies were observed, indicating photodamage to the photosystems. This can also be detrimental when mass cultures are grown under bright sunlight, when photoinhibition is even more pronounced and NPQ mechanisms are induced and waste absorbed light energy. Therefore, Perrine and coworkers constructed chlorophyllide a oxygenase (CAO) RNA interference (RNAi) mutants in *Chlamydomonas* to obtain transgenic strains with intermediate‐sized antenna complexes (20%–30% reduction in LHCII) instead of chl b‐less strains with total loss of the peripheral PSII antenna system (Perrine et al., 2012). The CAO RNAi mutants showed wild type‐like growth behavior under low light conditions but produced significantly more biomass (15%–35% compared to the wild type) when exposed to high light, without being impaired in photoprotective mechanisms, such as state transitions and xanthophyll‐dependent NPQ. However, one drawback is the reduced flexibility of these mutants to adjust antenna size to the prevailing conditions, especially outdoors, where light can show sharp dynamic fluctuations. To target this issue, the same authors improved their previous system by fusing the *CAO* gene to a light‐responsive transcription factor‐binding site of the *LHCMB6* gene and transferring this construct into a chl b‐less mutant background (Negi et al., 2020). With this system, the translational repressor NAB1 will bind to the *CAO* gene upon illumination and inhibit its expression, thus downregulating the LHCII antenna size in a light‐dependent fashion. These transgenic *Chlamydomonas* strains indeed showed highly dynamic adjustments of the antenna systems under fluctuating light conditions, with wild type‐like PSII quantum efficiencies, slightly lower NPQ and a three‐fold increase in biomass compared to control strains under dynamic light. It seems that this successful proof of concept may also have potential to improve productivity of crop species.

In crops, modifying light‐harvesting cross‐sections has led to a range of outcomes over the past 10 years. While soybean mutants with reduced chlorophyll levels did not show any increase in biomass accumulation when grown in the field (Slattery et al., 2017), a rice mutant expressing a maize GOLDEN2‐LIKE (GLK) transcription factor demonstrated enhanced chlorophyll and antenna complex biosynthesis, surprisingly resulting in 30%–40% more biomass and grain yields compared to wild type plants (Li et al., 2020). GLK proteins induce chloroplast development through regulation of plastid and nucleus‐encoded genes. Overexpression of GLK can even lead to the development of chloroplasts in nongreen tissues, such as roots. The resulting root photosynthesis of transgenic lines of the model plant *Arabidopsis thaliana* contributed to a small extent to overall CO_2_ assimilation in addition to leaf photosynthesis (Kobayashi et al., 2013). Nevertheless, the majority of studies focused on decreasing chlorophyll contents and antenna sizes (Song et al., 2017; Bielczynski et al., 2020) with positive outcomes for improving crop yields (Jin et al., 2016; Kirst et al., 2017; Friedland et al., 2019). While Jin and colleagues knocked out a chloroplast protein that regulates translation of chlorophyll biogenesis genes (HIGH PHOTOSYNTHETIC EFFICIENCY1, HPE1), Kirst and coworkers made use of an established yellow–green line with truncated light‐harvesting antennae in the model plant *Nicotiana tabacum* (tobacco) and observed higher biomass accumulation per unit absorbed light. Consequently, they proposed a shift in plant agronomy by reducing the space left between sowed plants in the field, as a strategy to increase the biomass outcome per field. Nevertheless, there is a fine balance between reducing the optical cross‐section of the LHC antennae for improved yields and preserving their functions in photoprotective mechanisms (Wu et al., 2020). It is also important to note that manipulation of the light‐harvesting antennae is easier to accomplish in unicellular/multicellular microalgae/cyanobacteria compared to highly differentiated, multiorgan systems, such as plants.

### Improving NPQ features increases biomass accumulation in dynamic light conditions

**Figure 6:**
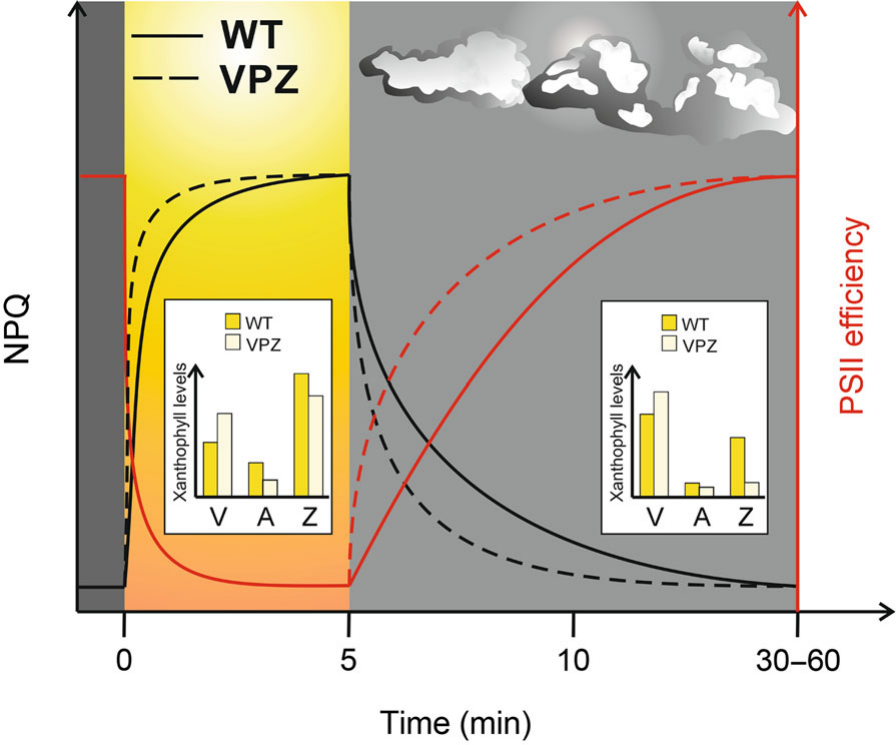
Activity of nonphotochemical quenching (NPQ) mechanisms and xanthophylls under changing light conditions and their potential for improving photosynthetic efficiency Plants often encounter sudden changes in light intensities (sunflecks or shading), which they quickly need to respond to and adjust their metabolic setup in order to avoid photodamage or maintain their photosynthetic capacity. Upon exposure to high light intensities, NPQ (black lines) components are swiftly initiated to dissipate excess excitation energy as heat and prevent photoinhibition of the photosynthetic machinery. Acidification of the thylakoid lumen initiates the fastest NPQ component qE (energy‐dependent quenching) within seconds to minutes, which is subsequently further enhanced through activation of the xanthophyll cycle, that is, the conversion of violaxanthin (V) into photoprotective zeaxanthin (Z) via antheraxanthin (A). Simultaneously, NPQ inhibits photosynthetic efficiency (red lines), which drops to very low levels under high light conditions. Upon the shift to low light or dark conditions, NPQ relaxes and pigment–protein photosystem II (PSII) efficiency recovers, observable through reconversion of zeaxanthin to violaxanthin. However, full relaxation of NPQ after high light stress is a rather slow process (30–60 min or longer), during which photosynthetic capacity is still inhibited to some extent under otherwise optimal conditions, thereby possibly losing time for biomass production. By overexpressing the lumenal pH sensor protein PsbS and the xanthophyll‐converting enzymes in tobacco (dashed lines), transgenic VPZ plants displayed faster NPQ relaxation under changing light conditions and thus faster recovery of photosynthesis, which resulted in higher biomass accumulation compared to control plants (Kromdijk et al., 2016). Graphs displayed here are schematic representations.

A complex component of photosynthesis is photoprotection of the electron transport chain from overexcitation under high light conditions. Photoprotective mechanisms are activated upon high light exposure to prevent overexcitation or dissipate excess absorbed light energy as heat (NPQ). Thus, in a quenched state, photosynthetic efficiency is downregulated until more favorable light conditions occur. However, upon shift from high light to low light conditions, NPQ mechanisms relax relatively slowly compared to their rate of induction, still inhibiting efficient photosynthesis for several minutes upon transition to more favorable light conditions (Zhu et al., 2004). During these minutes, valuable energy for photosynthetic biomass production is lost, which is particularly problematic under dynamic light conditions with short fluctuations in light intensities, as is the case in natural sunlight on a cloudy day for instance (Figure 6). Several studies have shown that plant growth is significantly reduced under fluctuating light conditions (Leakey et al., 2002; Kubásek et al., 2013; Vialet‐Chabrand et al., 2017; Kaiser et al., 2020). Scientists therefore aim to accelerate NPQ relaxation kinetics under fluctuating light through modification of the molecular players involved, in order to improve crop yields.

In an attempt to speed up NPQ relaxation kinetics in dynamic light conditions, Kromdijk and coworkers simultaneously overexpressed the PsbS protein and both xanthophyll cycle enzymes VDE and ZEP in tobacco (Kromdijk et al., 2016). Transgenic tobacco VPZ overexpression lines exhibited no significant differences from wild type tobacco plants when grown under steady state light conditions. However, under fluctuating light, VPZ lines outperformed control plants by 15%–20% increase in biomass under greenhouse as well as field‐grown conditions, owing to faster NPQ relaxation kinetics and improved net CO_2_ assimilation. Since these NPQ components are highly conserved in higher plants, this approach has great potential of increasing future crop yields. Nevertheless, expression of a VPZ construct in *Arabidopsis* did not result in the previously observed growth advantage in tobacco lines (Garcia‐Molina and Leister, 2020), demonstrating that different mechanisms might be at work and that cloning vector units might need optimization in different plant species. Furthermore, an overexpression line of PsbS in rice did also show about 20% increase in canopy radiation use efficiency into biomass and grain yield under fluctuating light (Hubbart et al., 2018). Modeling and 3D reconstruction of the rice canopy revealed that leaves in the lower canopy have higher capacities for photoprotective NPQ than leaves from the upper canopy (Foo et al., 2020), which may reflect the greater occurrence of fluctuations in light due to shading from the upper canopy and sunflecks. These results show the importance of considering the canopy context when trying to design strategies for photosynthetic improvement. In contrast to overexpressing PsbS in rice, tobacco plants overexpressing PsbS did not reveal any gain in biomass compared to control plants when grown under controlled and field conditions (Głowacka et al., 2018). However, these plants did display an increased water use efficiency of 25%–33% due to dampening of the light‐induced increase in stomatal conductance. Nevertheless, photosynthesis in general was not affected by PsbS overexpression. The authors proposed that PsbS abundance might modulate the redox state of the plastoquinone pool in the thylakoid membrane, which had been put forward as an early signal for stomatal opening in response to light (Busch, 2014). Whereas these studies exemplify the importance of the PsbS protein, there might be other genetic factors that could be utilized to adjust NPQ traits for improved crop performance. Quantitative genetics and genome‐wide association studies targeting natural variation in NPQ traits have already been done in soybean, rice, and *Arabidopsis*, revealing 15–33 putative new targets for manipulation of NPQ (Jung and Niyogi, 2009; Herritt et al., 2016; Wang et al., 2017; Rungrat et al., 2019).

A different avenue to explore could be the introduction of a different xanthophyll cycle into future crop plants. While the violaxanthin cycle is the predominant xanthophyll cycle in most tested plant species, a lutein epoxide cycle works in parallel to the violaxanthin cycle in some nonmodel plant species (Bungard et al., 1999). Epoxidation of lutein confers higher light‐harvesting efficiency and has been successfully engineered into *Arabidopsis* lines through expression of a ZEP gene from *Nannochloropsis oceanica* (Leonelli et al., 2017). Transgenic plants only expressing the lutein epoxide cycle displayed significantly higher PSII maximum quantum efficiencies and similar NPQ kinetics compared to plants employing the violaxanthin cycle, pointing at the potential of this alternative xanthophyll cycle for future studies.

All these different approaches have one thing in common—they require acidification of the thylakoid lumen, that is, a low pH through accumulation of protons. Lumen acidification is also accompanied by fluxes of counter‐ions over the thylakoid membrane, which help to maintain the balance between the ΔpH and the electric field (ΔѰ) component of the pmf. Influx of potassium ions (K^+^) into the lumen upon the shift from light to dark for instance increases ΔѰ, thereby allowing a decrease in ΔpH and the relaxation of the qE component of NPQ without compromising overall pmf to maintain ATP synthase activity (simulated by Davis et al., 2017). Therefore, counter‐ion fluxes could possibly be a novel target for modification of NPQ mechanisms (Davis et al., 2017). Indeed, Armbruster and coworkers demonstrated the significance of ion fluxes in adaptation to fluctuating light conditions by the action of the K^+^/H^+^ exchange antiporter KEA3, the protein family of which is important for pH and osmoregulation inside the chloroplast (Armbruster et al., 2014, 2016; Kunz et al., 2014; Correa Galvis et al., 2020; Li et al., 2021). In higher plants, there are three splice forms of KEA3, each of which has a distinct function, but generally they export protons from the lumen with concurrent import of potassium ions. Knockout of the *KEA3* gene in *Arabidopsis* resulted in a specific NPQ phenotype with higher NPQ levels upon the shift from dark to low light and slower NPQ relaxation from high to low light, which was attributed to the qE component of NPQ (Armbruster et al., 2014). Independent overexpression of the three KEA3 splice forms in the *KEA3* knockout background both in *Arabidopsis* and transiently in *Nicotiana benthamiana* showed the specific contribution of each isoform to this NPQ phenotype. KEA3.2 is the most abundant isoform and its overexpression led to lower NPQ levels than the wild type in the initial induction response in the transition from dark to low light, but not at high light, reflecting the opposite trends in NPQ kinetics of the *KEA3* knockout mutant. Thus, the oeKEA3.2 line also displayed significantly faster NPQ relaxation under fluctuating light through enhanced export of protons from the lumen. Overexpression of KEA3.3 had a similar low NPQ response to oeKEA3.2 in the shift from dark to low light. In contrast to oeKEA3.2 though, oeKEA3.3 also had a significantly lower NPQ amplitude in the transition to high light conditions, whereas oeKEA3.2 showed almost wild type levels during NPQ induction in high light despite its overexpression. These results indicate that KEA3.3 is more active than KEA3.2 in exporting protons from the lumen under high light stress, thus reducing and regulating overall NPQ levels. KEA3.2 and KEA3.3 splice forms distinguish by the presence of a KTN domain in KEA3.2, which is responsive to changes in NADH/NAD^+^ or ATP/ADP ratios or could possibly be regulated by the redox state of the plastoquinone pool (Armbruster et al., 2016). Overexpression of KEA3.1 did not show any special NPQ phenotype compared to the other isoforms and only slightly ameliorated the low/slow NPQ response of the *KEA3* knockout mutant, which exhibited a significantly slower growth rate when grown under fluctuating light. Surprisingly, no significant growth advantage of the overexpression lines could be detected, although oeKEA3.2 showed trends of enhanced growth. Further analyses revealed that KEA3 activity requires more fine‐tuning in order to optimize photosynthesis (Wang et al., 2017) and that combinations with other players involved in the generation of a ΔpH gradient over the thylakoid lumen (NDH complex in cyclic electron flow) can amplify the *KEA3* phenotype (Basso et al., 2020).

Many of the fore‐mentioned studies have not investigated the effects of ROS accumulation in the transgenic lines, but the manipulations are likely to have an impact on photo‐oxidative stress alleviation in addition to improved photosynthetic efficiencies (as suggested by Davis et al., 2017). Photo‐oxidative stress is established through the generation of the ROS singlet oxygen and superoxide, predominantly arising from PSII and PSI, respectively, through overreduction of the electron transfer chain. ROS mainly damage the core protein D1 of PSII, thereby downregulating photosynthesis under long‐term unfavorable conditions (photoinhibition). PSII employs an effective D1 repair cycle with *de novo* protein biosynthesis; however, this is a rather energy cost‐intensive process (Baena‐González and Aro, 2002). In attempts to engineer tobacco plants with enhanced resistance to drought stress, Almoguera and colleagues overexpressed the heat‐shock transcription factor A9 (HSFA9). This transcription factor was presumed to activate the expression of small heat‐shock proteins in chloroplasts and indeed conferred improved drought and oxidative stress tolerance as visible from sustained D1 protein levels after withholding of water (Almoguera et al., 2012). Similar results were achieved with an alternative approach in tobacco overexpressing a plastid‐encoded full‐length *PsbA* gene from maize under the control of the 35S promoter. These mutant lines had increased levels of the D1 protein and showed enhanced drought tolerance under stress conditions but wild type‐like growth phenotypes under optimal conditions (Huo et al., 2016). Following up on these studies, Chen and coworkers then combined and optimized both approaches and engineered a heat responsive PsbA construct expressed in the nucleus (Chen et al., 2020). The native *PsbA* gene is encoded in the chloroplast genome which is closer to the site of the PSII/D1 repair cycle for *de novo* synthesis upon recovery from photoinhibition. However, ROS production inside the chloroplast can strongly inhibit the translation of the PsbA messenger RNA (mRNA) into the D1 protein (Nishiyama et al., 2001, 2004). By transferring PsbA expression to the cell nucleus, D1 *de novo* synthesis could take place in the cytosol instead of the chloroplast. The mature protein was targeted to the chloroplast with the help of a chloroplast transit peptide (from RbcS) and remarkably, was able to replace degraded D1 protein in the PSII complex. Placing *PsbA* gene expression under the control of a heat responsive promoter from the HSFA2 (heat‐shock transcription factor A2) gene, enhanced gene expression upon exposure to increased temperatures. This presumably explained the enhanced heat stress tolerance in transgenic lines of *Arabidopsis*, tobacco, and rice, all of which also had significantly enhanced growth, biomass and grain yield both under nonstressed conditions and in field trials. Altogether, these results prove that photosynthesis can efficiently be upregulated through mitigation of photo‐oxidative stress and photoinhibition, leading to yield benefits in monocot as well as dicot plant species.

### Increasing electron transport capacity

Photosynthesis is composed of a series of electron transfer steps in the light reactions and enzymatic reactions in the Calvin–Benson–Bassham cycle. One of the major protein complexes within the thylakoid membrane besides the two photosystems is the cyt b6f complex, which is reduced and oxidized by the mobile electron carriers plastoquinol and plastocyanin, respectively, in LET between PSII and PSI. Via the Q‐cycle, the cyt b6f complex also transfers protons across the thylakoid membrane into the lumen in addition to its electron transfer function, thus contributing to the generation of a lumenal ΔpH gradient that fuels ATP synthase activity and the initiation of NPQ upon illumination. Electron flow through cyt b6f has long been proposed to be the key rate‐limiting step of the photosynthetic light reactions, since mutants of several plant species with inhibited cyt b6f expression showed downregulated photosynthetic capacity (Holloway et al., 1983; Price et al., 1995, 1998; Anderson et al., 1997; Ruuska et al., 2000; Yamori et al., 2011, 2016; Tikhonov, 2014). One of the eight cyt b6f subunits is the Rieske‐FeS protein, which is encoded in the nucleus and the expression of which determines the accumulation of the entire cyt b6f complex (Anderson et al., 1997; Price et al., 1998). Overexpressing this subunit both in a C3 (*Arabidopsis*, Simkin et al., 2017b) and a C4 (*Setaria viridis*, Ermakova et al., 2019) plant species gave rise to increased abundance of the cyt b6f complex and enhanced photosynthetic performance. In both studies, transgenic overexpression plants had higher PSII quantum efficiencies, lower NPQ values and higher CO_2_ assimilation rates than control plants. In contrast to *Arabidopsis* transgenic plants, *Setaria* overexpression lines were not reported to display any growth advantage over wild type plants, despite the 10% increase in CO_2_ assimilation. *Arabidopsis* overexpression lines, on the other hand, had 30%–70% more biomass and up to 50% greater seed yield compared to control plants. In both species, nevertheless, the abundance of the cyt b6f complex and the rate of CO_2_ fixation seem to be correlated through control of the electron transfer rate by cyt b6f. It will be interesting to see whether this crop improvement strategy could have similar outcomes in other C3 and C4 crop species in the future.

The next step within photosynthetic light reactions downstream of the cyt b6f complex involves electron transfer through the thylakoid lumen toward PSI via the soluble protein plastocyanin, which is encoded by two genes (*PetE*) in higher plant genomes. This electron carrier has been shown to be essential for light‐dependent electron transfer as a double knockout mutant of both *PetE* genes is only viable when grown heterotrophically on media containing sucrose, but not when grown on soil only (Weigel et al., 2003). Therefore, plastocyanin‐mediated electron transfer might be a rate‐limiting step in higher plant photosynthesis as well (Burkey, 1994; Burkey et al., 1996; Schöttler et al., 2004; Finazzi et al., 2005; Höhner et al., 2020), although even low levels of this protein seem capable of efficiently sustaining photosynthetic light reactions (Abdel‐Ghany, 2009; Pesaresi et al., 2009). Attempts have been made to overexpress plastocyanin genes in *Arabidopsis*, which indeed resulted in enhanced biomass production of up to 1.6‐fold compared to the wild type, even though photosynthetic parameters were similar to wild type levels and no improvement of photosynthetic capacity could be observed (Pesaresi et al., 2009). This phenomenon could possibly be explained with the newly discovered role of plant plastocyanins in oxidative stress tolerance (Zhou et al., 2018). Plastocyanins are copper‐binding proteins and expression of the *PetE* genes is highly dependent on copper availability. While PetE2 is the predominant plastocyanin expressed under nonstress and copper‐enriched conditions and acts as a buffer in copper homeostasis, PetE1 expression increases upon copper starvation and functionally replaces PetE2 (Abdel‐Ghany, 2009). Under stress conditions, copper is released into the chloroplast via two P‐type ATPases (Abdel‐Ghany et al., 2005) and reacts with H_2_O_2_ in a Fenton reaction, generating the highly reactive ROS hydroxyl radical (Sutton and Winterbourn, 1989). Through introduction of the *PetE2* gene from the halophytic plant *Suaeda salsa* (Song and Wang, 2014) into *Arabidopsis*, a plastocyanin with a greater copper‐binding capacity was able to confer a greater tolerance of transgenic *Arabidopsis* lines to oxidative stress than the original *Arabidopsis* plastocyanins (Zhou et al., 2018). This resulted in stress‐tolerant *Arabidopsis* plants with a fresh weight three to four times higher than that of wild type plants grown under ROS producing stress conditions. It is, therefore, of high importance to explore natural variation of protein features, especially in plant species living in extreme environments and to exploit their potential of being translated into agricultural crop species. In addition, it may also be important to look further than just proteins for the regulation of photosynthetic processes. Other regulatory elements, such as microRNAs, for post‐transcriptional gene expression regulation have gained more and more interest in the past 20 years (Meyers and Axtell, 2019; Wang et al., 2019). So far, no microRNA has been discovered that directly controls plastocyanin gene expression. However, through an indirect mechanism, one of the microRNAs with high conservation in plants, miR408, was found to impact copper levels inside the chloroplast through gene regulation of two copper transporters in the chloroplast envelope and thylakoid membranes. Overexpression of miR408, hence, led to increased expression of plastocyanin as well as other photosynthetic genes, resulting in enhanced biomass accumulation of 10%–20% in *Arabidopsis*, tobacco, and rice plants (Pan et al., 2018). Interestingly, this approach not only yielded improved vegetative plant growth but also boosted seed and grain size and weight even under field‐grown conditions. Moreover, miR408 seems to be highly conserved in eudicot and monocot plant species, making it a promising target for improved crop growth.

The last step of LET encompasses the protein FNR, which accepts electrons from Fd and subsequently reduces NADP^+^ to NADPH. Efforts have been made to overexpress FNR in tobacco, but no growth phenotype could be observed despite an increase in oxidative stress tolerance (Rodriguez et al., 2007). Knockdown of FNR, on the other hand, drastically decreased the mutants' photosynthetic capacity and made it highly susceptible to oxidative stress (Lintala et al., 2012). However, recently it has been proposed that the interaction between FNR and the Thylakoid RhOdanase‐Like protein (TROL), the protein that putatively binds FNR to the thylakoid membrane, could have potential for crop improvement (Fulgosi and Vojta, 2020). TROL is a membrane‐spanning protein close to PSI in the grana margins, with a rhodanase‐like domain (RHO) exposed to the thylakoid lumen. Here, the RHO domain may be involved in sensing redox signals, upon which FNR‐binding on the stromal side to the membrane recruiting motif (MRM) of TROL is adjusted, possibly in a light‐ and pH‐dependent manner. It is postulated that FNR is membrane‐bound in the dark, reversing its function and providing reduced Fd to a number of metabolic pathways, including oxidative stress tolerance. Upon light exposure, FNR is released into the stroma, only then being active in LET toward NADPH regeneration. Hence, TROL could potentially be a target for the switch in FNR action mode, either through modifications of the redox sensor domain RHO or the FNR‐binding domain MRM.

Instead of forwarding electrons to FNR, Fd can also reduce components of AET routes to stimulate ATP synthesis under stress conditions. However, the exact mechanisms are not yet clear and contrasting views on the function of these AET complexes as alternative electron acceptors downstream of PSI have recently been brought forward (Nawrocki et al., 2019a; Buchert et al., 2020; Rantala et al., 2020; Zhou et al., 2020; Rühle et al., 2021; Wu et al., 2021). Nevertheless, it is well‐accepted that the energy balance is of high importance when it comes to altering metabolic processes (Kramer and Evans, 2010). Reducing the levels of PGR5 protein seems to negatively impact plant growth to some extent under fluctuating light conditions (Munekage et al., 2008; Nishikawa et al., 2012), whereas in *Chlamydomonas*, deletion of this protein promotes biotechnological hydrogen production due to rerouting of electrons toward the hydrogenase HydA upon stress induction (Steinbeck et al., 2015; Nagy et al., 2021). Relative to C3 species, C4 plant species rely more strongly on CET around PSI in bundle sheath cells in order to provide extra ATP to fuel the carbon concentrating mechanism. However, although overexpression of PGR5 in *Flaveria bidentis* led to alleviation of PSI acceptor side limitation, it did not result in enhanced CO_2_ fixation (Munekage et al., 2010; Tazoe et al., 2020). In contrast, overexpression of PGR5 resulted in increased growth rates in diatoms under fluctuating light (Zhou et al., 2021) and enhanced high light and drought stress in *Arabidopsis* (Long et al., 2008). In addition, PTOX is also important as an alternative electron sink and for chloroplast biogenesis and carotenoid biosynthesis during early leaf development, and induction of its expression under stress conditions (summarized in Sun and Wen, 2011 and Johnson and Stepien, 2016; Ivanov et al., 2012; Li et al., 2016a; Ghotbi‐Ravandi et al., 2019) led to the suggestion that it could be a suitable target for enhancing stress tolerance in plants (Johnson and Stepien, 2016). However, initial experiments overexpressing PTOX in *Arabidopsis* and tobacco did not result in enhanced tolerance to high light stress but instead made plants more photosensitive (Joët et al., 2002; Rosso et al., 2006; Heyno et al., 2009; Ahmad et al., 2012, 2020). Nevertheless, overexpression of PTOX did provide an advantage when exposed to salt stress. This effect seemed to rely on a translocation of PTOX from the stroma lamellae to the appressed grana stacks in salt‐tolerant plants (Stepien and Johnson, 2018; Ahmad et al., 2020). This correlation between PTOX expression and salt stress tolerance was also recently confirmed in a halophyte C4 plant species (Essemine et al., 2020).

Instead of indirectly upregulating the production of ATP through upregulation of CET routes, it is conceivable to directly enhance chloroplast ATP synthase activity (Cardona et al., 2018; Davis and Kramer, 2020). The ATP synthase is composed of two rotary motor complexes (CF0/CF1) which are connected by two flexible stalks (Kühlbrandt, 2019). The CF0 complex is embedded within the thylakoid membrane and consists of a ring of several c‐subunits, the numbers of which are organism/species‐dependent but remain constant under different conditions (between eight and 17 c‐subunits; Davis and Kramer, 2020). Each c‐subunit binds one proton from the thylakoid lumen, fueling the CF0 motor and subsequently the CF1 motor on the stromal side of the membrane, thus promoting the production of three ATP molecules per 360° rotation. Davis and Kramer (2020) recently proposed a theoretical model from kinetic simulations of photosynthetic reactions that considers the size of the c‐ring/number of c‐subunits per ring and therefore the ratio of required protons per generated ATP. A lower ratio (a smaller c‐ring size) would theoretically result in higher photosynthetic energy conversion rates. However, the simulations predicted that a smaller ring size would also lead to a higher ΔpH across the membrane, thus activating photoprotective NPQ mechanisms and limiting photosynthetic efficiencies even under dark conditions. Evolution has therefore favored a bigger ring size instead, in order to avoid photodamage at the cost of conversion efficiencies. Nevertheless, the concept of optimizing the H^+^/ATP ratio (Pogoryelov et al., 2012) could provide a novel avenue for improving photosynthesis, considering further adjustments to the photosynthetic machinery. In a different approach, mutant lines with point mutations in ATP synthase genes were analyzed in different organisms. In the cyanobacterium *Synechococcus elongatus* sp. PCC 7942, a single nucleotide polymorphism (SNP) was identified in the *atpA* gene, which enhanced ATP synthase contents and activity, conferring improved photosynthetic efficiency as well as carbon fixation rate, especially under stress conditions (Lou et al., 2018). Certain point mutations in the *AtpB* gene of the CF1 complex, on the other hand, appear to be deleterious for the assembly of the entire complex and normal plant growth, so that mutants spontaneously reverted to the wild type gene sequence (Robertson et al., 1989; Malinova et al., 2021). Additionally, a SNP of a threonine residue in the β‐subunit of CF1 (T86A in *AtpB*) was identified in cold‐tolerant cucumber species when the gene sequences were compared to cold‐susceptible species. Threonine residues are often subject to post‐translational modification with regulatory phospho groups. Lack of a phosphorylation site in *AtpB* due to this SNP could possibly change its mode of action and therefore confer improved tolerance to cold stress (Oravec and Havey, 2021). Furthermore, an *Arabidopsis* mutant line with altered ATP synthase regulation was isolated from an ethyl methanesulfonate library, revealing a point mutation in the γ1‐subunit of the central stalk (Wu et al., 2007; Kanazawa et al., 2017). This mutation resulted in about 50% loss of ATP synthase protein content without compromising its overall activity under low light conditions when compared to the wild type, indicating a higher activity of the remaining complexes in the mutant. However, when exposed to stressful conditions, such as low CO_2_ and fluctuating light, the mutant was much more susceptible to photoinhibition. The authors concluded that this particular protein residue is involved in the stress‐related downregulation of the ATP synthase activity, rendering a fraction of the ATP synthase pool inactive to prevent overreduction of the electron chain (Kanazawa et al., 2017). These results show that it is not sufficient to simply aim for constantly enhanced ATP synthase activity, but that it is necessary to consider its regulatory mechanisms under suboptimal conditions as well to maintain the plant's capacity for the induction of NPQ and a healthy ATP to NADPH ratio. Such control mechanisms do not only act on the membrane‐bound CF0 complex through the pmf but are also administered through thiol‐based redox regulation of cysteine residues on the γ‐subunit of the CF1 motor (Yang et al., 2020; Buchert et al., 2021).

Thioredoxins are proteins that reduce disulphide bonds of cysteines in a light‐dependent manner, thus regulating protein activity (Nikkanen and Rintamäki, 2019). A chloroplastic NADPH‐dependent thioredoxin reductase C (NTRC) is known to interact with the ATP synthase γ‐subunit (Nikkanen et al., 2016). Overexpression of NTRC in *Arabidopsis* resulted in significantly enhanced biomass accumulation, starch production, photosynthetic efficiency, NDH‐dependent CET and photo‐oxidative, drought and heat stress tolerance compared to the wild type, especially under low light conditions, which was mainly attributed to lower acceptor side limitation of PSI (Chae et al., 2013; Toivola et al., 2013; Nikkanen et al., 2016, 2018; Kim et al., 2017). Interestingly, the overexpression mutant also had lower NPQ under light‐limiting conditions up to about 500 µmol photons/m^2^/sec and displayed much faster NPQ relaxation upon high to low light transitions under fluctuating light conditions (Guinea Diaz et al., 2020). However, NTRC overexpression in tobacco resulted in a slight growth retardation during early plant development when compared to the wild type despite higher leaf starch content (Ancín et al., 2019). The increased starch content was suggested to derive from decreased starch turnover during the night rather than enhanced starch biosynthesis during the day. A connection between redox regulation and starch metabolism is present in a range of plant species and organs (Sanz‐Barrio et al., 2013; Hou et al., 2019) and might have potential for future bioengineering of starchy crops (Nikkanen et al., 2017).

### Translating strategies from lower plants/microalgae/cyanobacteria into higher plants

Throughout evolution, oxygenic photosynthesis has developed in cyanobacteria first and subsequently in microalgae and plants upon endosymbiotic events with heterotrophic eukaryotes. Different photosynthetic organisms had to adjust to different environmental conditions, depending on the prevalent light and CO_2_ levels. Many years of basic research have revealed different traits and mechanisms and variations in protein complexes between aquatic and terrestrial species that have been optimized to adjust to certain stress conditions. It is, therefore, of high interest to translate this basic knowledge of possibly more effective ancestral proteins and potentially advantageous mechanisms from lower organisms into higher plants to ultimately improve photosynthetic performance in crops. Successful examples of this concept include the expression of cyanobacterial/algal Calvin cycle enzymes in several plant and crop species (recently reviewed in Simkin et al., 2019).

One success story has been the introduction of the algal cytochrome c6 (cyt c6) protein into *Arabidopsis* and tobacco, which improved photosynthetic electron transfer and biomass accumulation even under field conditions (Chida et al., 2007; Yadav et al., 2018; López‐Calcagno et al., 2020). This soluble protein is present in many cyanobacteria and green algae species and is located in the thylakoid lumen where it shuttles electrons between the cyt b6f complex and PSI for light‐dependent LET of photosynthesis, in the same fashion plastocyanin operates, as well as between cyt b6f and terminal oxidases for respiration in cyanobacteria (Torrado et al., 2019; Viola et al., 2021). Cyt c6 and plastocyanin expression levels are dependent on iron and copper availability, respectively, (García‐Cañas et al., 2021) as plastocyanin binds copper, whereas cyt c6 is an iron‐binding protein that seems to have been lost in green plants after the Great Oxidation Event (recently reviewed in Castell et al., 2021b and Slater et al., 2021). When the atmosphere became enriched in oxygen, which readily reacts with iron, the level of available iron ions as co‐factors for cyt c6 was limited, thus promoting the activity of plastocyanin in green plants instead. In cyanobacteria, there are two more cyt c6 gene homologs present, which were proposed to have arisen from cyt c6 gene duplications and were annotated as cyt c6B. Interestingly, green plants lost the cyt c6 and cyt c6B genes but instead contain a gene homolog, cyt c6A, which likely evolved from cyt c6B through insertion of a loop insertion peptide (Slater et al., 2021). However, both cyt c6A and c6B proteins show much lower redox midpoint potentials compared to cyt c6 and are, therefore, not likely to contribute to electron transfer in the thylakoid lumen, in contrast to cyt c6 (Molina‐Heredia et al., 2003; Bialek et al., 2014). It has been demonstrated that cyt c6 proteins from different algae/seaweed species are suitable for introduction into plant model species to effectively perform electron transfer, having been selected based on a similar redox midpoint potential to plant plastocyanins. While Chida and coworkers inserted a cyt c6 gene from the red alga *Porphyra yezoensis* into *Arabidopsis* (Chida et al., 2007), Yadav and coworkers utilized a cyt c6 gene from the green macroalga *Ulva fasciata* (sea lettuce) and introduced it into tobacco (Yadav et al., 2018). In both cases, the algal genes were under the control of the constitutive cauliflower mosaic virus 35S (CaMV35S) promoter and were fused to a plant species‐specific *PetE* transit peptide for correct localization of the cyt c6 protein into the chloroplast thylakoid lumen. Both studies reported enhanced growth phenotypes during the first 8 weeks of plant growth, in accordance with increased chlorophyll and photosynthetic metabolite contents, although other photosynthetic parameters were only slightly improved. An even more advanced approach was recently undertaken by López‐Calcagno and coworkers by combining enhancements of LET and carbon fixation in two tobacco cultivars through addition of an algal cyt c6 gene from *Porphyra umbilicalis* and the bifunctional cyanobacterial FBP/SBPase from *Synechocystis* sp. PCC 7942 or *SBPase* gene from higher plants, respectively (López‐Calcagno et al., 2020). While the single mutants had slightly increased photosynthetic rates, the double mutants showed significantly enhanced CO_2_ assimilation rates (up to 15% more than control plants) and PSII operational efficiencies. Consequently, single mutants had 9%–44% and double mutants had 32%–52% more biomass than control plants when grown in a controlled environment in a glasshouse. Field experiments with these mutants, on the other hand, showed much more variation in terms of biomass gains. During a small field trial in 2016, leaf material from single mutants was harvested before the flowering stage and again displayed a 20%–44% increase in biomass accumulation. However, in the following field season only a small growth advantage of the double mutants compared to control plants was visible when plant material was harvested after the onset of flowering. Surprisingly, photosynthetic parameters were not significantly different from control plants, although an improved intrinsic water use efficiency could be measured. Nevertheless, these mutant constructs targeting both the light reactions as well as carbon fixation seem to have great potential for improving crop yields, especially for plant species with short generation times. In addition, it was also demonstrated that introduction of a plastocyanin gene from *Chlamydomonas* into the diatom *Phaeodactylum tricornutum*, which normally only contains cyt c6, can enhance biomass production under iron‐deficient conditions by 60% when compared to the wild type (Castell et al., 2021a). All these studies show that it would be indeed beneficial for photosynthetic organisms to employ both soluble electron carriers plastocyanin and cyt c6 since either protein can functionally replace the other one when nutrients are short of either iron or copper ions, respectively, leaving room for biotechnological improvements of crop yields.

A similar story to plastocyanin and cyt c6 can be told about the Fd and flavodoxin proteins in plants and cyanobacteria, respectively. Fd is a conserved FeS electron carrier in cyanobacteria and plants, whereas flavodoxin binds flavin mononucleotide as cofactor and was lost from plants and green algae during the course of evolution due to its functional redundancy. In contrast to plastocyanin/cyt c6, no definite answer has been found yet as to why the iron‐containing Fd has survived the Great Oxidation Event and has been maintained in plants, where flavodoxin has not (Pierella Karlusich et al., 2014). In the past 15 years, many studies on the expression of a cyanobacterial flavodoxin in plants have been published. Most of these studies reported on enhanced tolerance of transgenic plants to different environmental stresses, particularly iron deficiency and oxidative stress, under which Fd levels would normally decrease but can now be compensated for by flavodoxin expression (Tognetti et al., 2006, 2007a, 2007b; Zurbriggen et al., 2008; Shvaleva et al., 2009; Coba de la Peña et al., 2010; Ceccoli et al., 2012; Lodeyro et al., 2012; Gharechahi et al., 2015; Mayta et al., 2018; Gómez et al., 2020; Niazian et al., 2021). It was also shown that flavodoxin could functionally replace Fd in plants (Blanco et al., 2011) and additional expression of a cyanobacterial FNR could increase oxidative stress tolerance even more (Giró et al., 2011). Flavodoxin expression is not always beneficial and may lead to stunted plant growth (Li et al., 2016b; Mayta et al., 2019) although detrimental effects on yield were offset by a higher harvest index compared to wild type plants (Mayta et al., 2019). Overall, flavodoxin expression in agricultural crops seems to have great potential to enhance abiotic stress tolerance.

Another class of photosynthetic flavoproteins that disappeared in flowering plants (angiosperms) throughout evolution are flavodiiron proteins (FDPs). FDPs serve as photoprotective excess electron valves in the so‐called “Mehler‐like reaction” or water–water cycle of photosynthesis (Allahverdiyeva et al., 2015; Ilík et al., 2017; Alboresi et al., 2019) across a large part of the green lineages from cyanobacteria up to gymnosperms. In these organisms, electron transfer on the acceptor side of PSI, initiated by the splitting of water at PSII, can switch from FNR and the regeneration of NADPH to FDPs in an alternative pathway under stress conditions. When the electron transfer chain becomes over‐reduced, FDPs help to release excess electron pressure downstream of PSI by reducing molecular oxygen to water, thereby closing the water–water cycle and preventing the generation of the highly reactive ROS superoxide for photoprotection of PSI. In cyanobacteria, FDPs are divided into two clusters and either work as homodimers (Mustila et al., 2016) or heterodimers composed of one FDP of each cluster (Santana‐Sanchez et al., 2019). The heterodimer Flv1/3 is conserved in all cyanobacteria and is responsible for oxygen photoreduction downstream of PSI in a Mehler‐like reaction that does not produce ROS (Allahverdiyeva et al., 2013), whereas the heterodimer Flv2/4 is only present in β‐cyanobacteria and was reported to be involved in photoprotection of both PSII and PSI (Zhang et al., 2009, 2012; Bersanini et al., 2014, 2017; Chukhutsina et al., 2015; Santana‐Sanchez et al., 2019). In angiosperms, in which FDPs are absent, introduction of two FDPs could therefore possibly replace several ROS scavenging enzymes and reactions, thus saving energy and nitrogen sources or adding extra protection. Indeed, transgenic lines of tobacco, *Arabidopsis* and barley expressing cyanobacterial Flv1/3 or Flv2/4 proteins in chloroplasts showed that FDPs are able to act as additional electron sinks in plants as well, particularly under stress conditions, such as drought and fluctuating light stress, thereby improving photosynthetic performance (Gómez et al., 2018; Tula et al., 2020; Shahinnia et al., 2021; Vicino et al., 2021). In other instances, two FDPs from the moss *Physcomitrella patens* were introduced into *Arabidopsis* and rice, revealing similar effects of FDPs and CET in maintaining the pmf under unfavorable growth conditions (Yamamoto et al., 2016; Wada et al., 2018). In summary, both sets of FDPs seem to fulfill similar roles when expressed in angiosperms, even though they were proposed to have different photoprotection targets (PSI vs. PSII) in cyanobacteria. In one case, transgenic plants even displayed improved biomass accumulation under nonstress conditions (Tula et al., 2020), suggesting that FDPs are promising tools for bioengineering of future crops (Mullineaux, 2016).

Further cyanobacterial systems with potential for improving photosynthetic efficiencies in plants are currently under investigation and include the OCP and novel chlorophylls with absorption wavelengths in the far‐red spectrum. These red‐shifted chlorophylls d and f with absorption maxima at 740 and 760 nm, respectively, have been discovered in certain cyanobacterial species (Li and Chen, 2015). The novel chlorophyll d is predicted to be able to bind to LHC proteins, expanding the range of light absorption of chlorophyll a beyond 700 nm into the far‐red region, which is often found in the lower canopy of plants and could therefore enhance light harvesting and boost crop yields (Elias et al., 2021). Cyanobacterial OCP, on the other hand, absorbs wavelengths in the blue–green region and is attached to the light‐harvesting antennae (phycobilisomes), where it is responsible for photoprotective dissipation of excess light energy as heat (NPQ) and scavenging of ROS (Muzzopappa and Kirilovsky, 2020). This photoreceptor protein consists of an effector N‐terminal domain (NTD), a sensor C‐terminal domain (CTD), and one ketocarotenoid (3‐hydroxyechinenone, echinenone, canthaxanthin) or the xanthophyll zeaxanthin. Upon absorption of strong blue–green light, the noncovalently bound ketocarotenoid transfers from the CTD to the NTD, thereby undergoing a conformational change and a shift in color from orange to red, thus activating the quenching state. OCP has not only been expressed in ketocarotenoid‐producing microalgae for better solubilization of the ketocarotenoids canthaxanthin and astaxanthin for their use as nutraceuticals (Pivato et al., 2021), but is also being exploited as a possible photoswitchable protein in plants with implementations for plastid optogenetics, artificial photosynthesis and synthetic biology due to its light‐dependent conformational changes and uniqueness in cyanobacteria (Andreoni et al., 2017; Lechno‐Yossef et al., 2017; Dominguez‐Martin and Kerfeld, 2019; Piccinini et al., 2021).

## CONCLUSIONS

**Figure 7:**
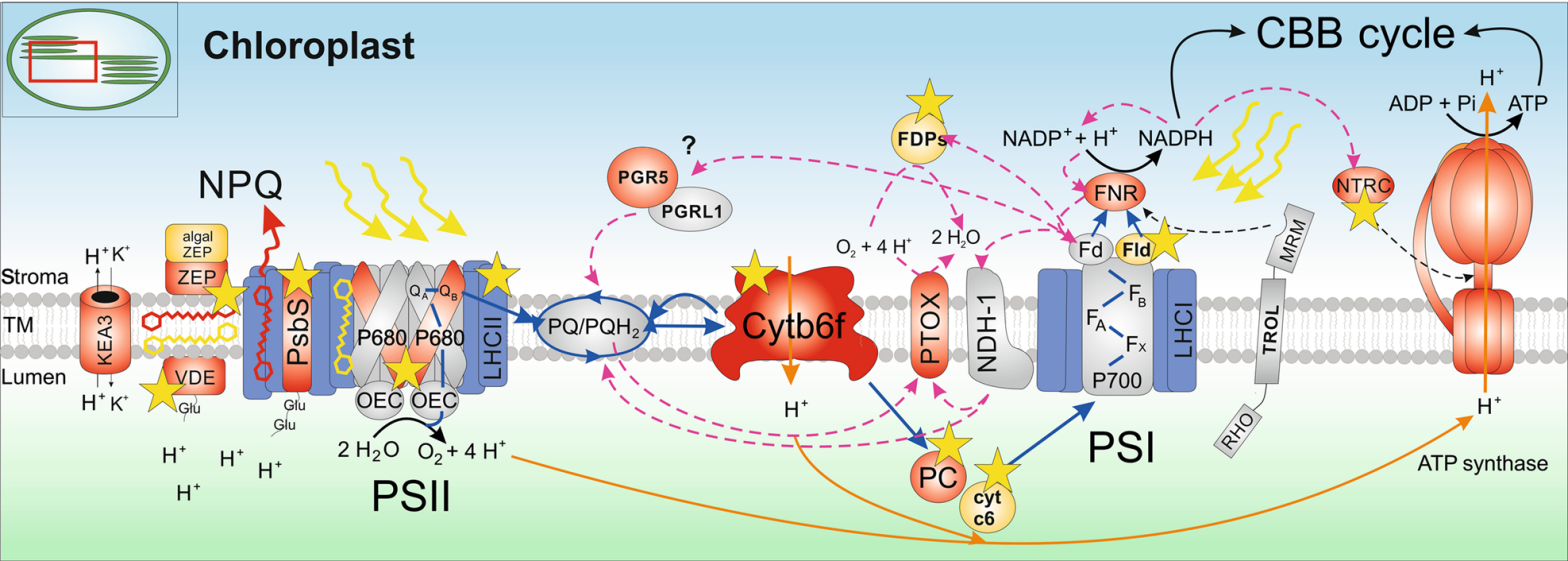
Schematic overview of light reaction components that have been targeted for bioengineering improved crops Strategies for boosting plant photosynthesis through manipulations of light reaction components are highlighted in either red or blue in this depiction of Figure 3. Components that have not been investigated further yet are depicted in gray. Proteins and protein complexes that are highlighted in red have been directly overexpressed or their expression has been indirectly induced and resulted in a measurably improved phenotype compared to control plants. Blue highlighting of protein complexes indicates downregulation of these components. Yellow–orange proteins symbolize the introduction of alternative pathways deriving from lower plants, microalgae and cyanobacteria, including an algal zeaxanthin epoxidase (ZEP), cytochrome c6 (cyt c6), flavodoxin (FLD), and flavodiiron proteins (FDPs). Phenotypes were either associated with higher biomass accumulation (labeled with yellow asterisks) or enhanced abiotic stress tolerance (see also Table 1). For further explanation of the light reactions see the original Figure 3.

“Photosynthesis: Ancient, essential, complex, diverse… and in need of improvement in a changing world” (Niinemets et al., 2016). This title of a conference summary article neatly describes the need for a shift in understanding photosynthesis and its potential applications. Photosynthetic organisms have optimized photosynthesis according to their needs, which includes survival and reproduction rather than enhanced yields. Modern techniques of bioengineering, through synthetic biology and gene editing, have provided useful means to targeting specific features of metabolic pathways in a timelier manner than conventional breeding does and are rapidly gaining traction in re‐engineering of photosynthesis (Zhu et al., 2020). Our review has highlighted research demonstrating that it is possible to improve photosynthetic electron transport (Figure 7; Table 1). Targeting the light‐harvesting antenna size is a high potential approach in both plants as well as photosynthetic micro‐organisms, with a three‐fold increase in microalgae biomass (Negi et al., 2020) and 25%–40% more plant biomass in transgenic tobacco and *Camelina*, respectively (Kirst et al., 2017; Friedland et al., 2019). Furthermore, improving photoprotective traits, such as NPQ relaxation and D1 photoprotection, resulted in 15%–20% enhanced growth in field‐grown plants (Kromdijk et al., 2016; Hubbart et al., 2018; Chen et al., 2020). LET reactions are very well studied and gene expression manipulation of the Rieske‐FeS protein of the Cyt b6f complex and the mobile electron carrier plastocyanin were revealed as the most promising targets for boosting plant yields (Simkin et al., 2017b; Pan et al., 2018). AET pathways in higher plants, on the other hand, are less well understood, and manipulation of the putative components seems to affect stress tolerance rather than enhance photosynthetic efficiency. In contrast, introduction of alternative electron carriers and acceptors from lower plants, microalgae and cyanobacteria, which often show higher efficiencies than their equivalents in higher plants, could restore electron transfer pathways that were lost in higher plants during the course of evolution and improve plant growth (cyt c6: Chida et al., 2007; Yadav et al., 2018; López‐Calcagno et al., 2020; FDPs: Tula et al., 2020). In addition to the strategies discussed, combinations of different approaches may be strongly synergistic and when translated into staple crop species may allow an even greater boost in photosynthetic efficiency and crop productivity.

**Table 1:** Overview of different approaches used to improve light reactions

Strategy and gene of interest	Targeted process or protein complex	Species	Phenotype associated with increased biomass accumulation	Phenotype associated with enhanced abiotic stress tolerance	References
Introduction of a maize GOLDEN2‐LIKE (GLK) transcription factor for overexpression of photosynthetic genes	LHCs	Rice	30%–40%	—	Li et al., 2020
Knockout of HIGH PHOTOSYNTHETIC EFFICIENCY1 (HPE1) for impaired chlorophyll biogenesis	LHCs	*Arabidopsis*	Yes	—	Jin et al., 2016
Truncated light‐harvesting antennae mutant	LHCs	Tobacco	Yes	—	Kirst et al., 2017
RNAi of CAO for reduced chlorophyll b expression	LHCs	*Camelina sativa*	40%	—	Friedland et al., 2019
Overexpression of PsbS, VDE and ZEP	NPQ	Tobacco	15%–20%	—	Kromdijk et al., 2016
Overexpression of KEA3	NPQ	*Arabidopsis* and tobacco	Slightly	—	Armbruster et al., 2016
Overexpression of HSFA9 for D1 protection	Photoprotection	Tobacco	—	Drought stress	Almoguera et al. 2012
Overexpression of maize PsbA	Photoprotection	Tobacco	—	Drought stress	Huo et al., 2016
Engineering a heat responsive PsbA construct expressed in the nucleus	Photoprotection	*Arabidopsis*, tobacco, rice	Yes (also under nonstress conditions)	Heat stress	Chen et al., 2020
Overexpression of the Rieske‐FeS protein	cyt b6f	*Arabidopsis, Setaria viridis*	30%–70% (*Arabidopsis*)	—	Simkin et al., 2017b
			10 increased CO_2_ assimilation rate in *Setaria*		Ermakova et al., 2019
Overexpression of plastocyanin	Electron transfer	*Arabidopsis*	1.6‐fold	—	Pesaresi et al., 2009
Overexpression of PetE2 from *Suaeda salsa*	Electron transfer by plastocyanin	*Arabidopsis*	3–4‐fold	Oxidative stress	Zhou et al., 2018
Overexpression of miR408 for enhanced copper uptake into the chloroplast	Electron transfer by plastocyanin	*Arabidopsis*, tobacco, rice	10%–20%		Pan et al., 2018
Overexpression of FNR	Electron transfer	Tobacco	—	Oxidative stress	Rodriguez et al., 2007
Overexpression of (algal) PTOX	AET	*Arabidopsis, Eutrema salsugineum*, tobacco	—	Salt stress	Stepien and Johnson 2018; Ahmad et al., 2020
SNPs in *atpA* for enhanced ATP synthase levels	ATP synthesis	*Synechococcus elongatus* sp. PCC 7942	—	Heat stress	Lou et al., 2018
SNPs in the β‐subunit of CF1	ATP synthesis	Cucumber	—	Cold stress	Oravec and Havey 2021
Overexpression of NTRC	Redox regulation	*Arabidopsis*, tobacco	Yes	Oxidative/drought/heat stress	Chae et al., 2013; Toivola et al., 2013; Nikkanen et al., (2016, 2018; Kim et al., 2017; Ancín et al. 2019
Overexpression of algal cyt c6	Electron transfer	*Arabidopsis*, tobacco	Up to 50%	—	Chida et al., 2007; Yadav et al., 2018; López‐Calcagno et al., 2020
Overexpression of cyanobacterial flavodoxin	Electron transfer	Tobacco	—	Iron deficiency, oxidative stress	Tognetti et al., 2006, 2007a, 2007b; Zurbriggen et al., 2008; Shvaleva et al., 2009; Coba de la Peña et al. 2010; Giró et al., 2011; Ceccoli et al., 2012; Lodeyro et al., 2012; Gharechahi et al., 2015; Mayta et al., 2018; Gómez et al., 2020; Niazian et al., 2021
Overexpression of cyanobacterial and moss FDPs	AET	*Arabidopsis*, tobacco, barley, rice	Yes	Drought/fluctuating light stress	Yamamoto et al., 2016; Gómez et al., 2018; Wada et al., 2018; Tula et al., 2020; Shahinnia et al., 2021; Vicino et al., 2021

Abbreviations: AET, alternative electron transfer; ATP, adenosine triphosphate; LHC, light‐harvesting complex; NPQ, nonphotochemical quenching.

## CONFLICTS OF INTEREST

The authors declare there are no conflicts of interest.
